# Alcohol use and associated risk factors among female sex workers in low- and middle-income countries: A systematic review and meta-analysis

**DOI:** 10.1371/journal.pgph.0001216

**Published:** 2023-06-13

**Authors:** Alicja Beksinska, Oda Karlsen, Mitzy Gafos, Tara S. Beattie

**Affiliations:** Department of Global Health and Development, Faculty of Public Health and Policy, London School of Hygiene and Tropical Medicine, London, United Kingdom; Bangladesh University of Health Sciences, BANGLADESH

## Abstract

Due to its widespread use in the sex work industry, female sex workers (FSWs) in low- and middle-income countries (LMICs) are at high risk of harmful alcohol use and associated adverse health outcomes. Factors associated with harmful alcohol use include violence, mental health problems, drug use, sexual risk behaviors and HIV/STIs. To our knowledge, there has been no quantitative synthesis of FSW alcohol use data to date. This systematic review and meta-analysis aims to provide an estimate of the prevalence of harmful alcohol use among FSWs in LMICs and to examine associations with common health and social concerns. The review protocol was registered with PROSPERO, number CRD42021237438. We searched three electronic databases for peer-reviewed, quantitative studies from inception to 24th February 2021. Studies were selected for inclusion that reported any measure of prevalence or incidence of alcohol use among FSWs aged 18 or older from countries defined as LMIC in accordance with the World Bank income groups 2019. The following study designs were included: cross-sectional survey, case–control study, cohort study, case series analysis, or experimental study with baseline measures for alcohol use. Study quality was assessed with the Center for Evidence-Based Management (CEBMa) Critical Appraisal Tool. Pooled prevalence estimates were calculated for (i) any hazardous/harmful/dependent alcohol use, (ii) harmful/dependent alcohol use only, both overall and by region and (iii) daily alcohol use. Meta-analyses examined associations between harmful alcohol use and violence, condom use, HIV/STIs, mental health problems and other drug use. In total, 435 papers were identified. After screening, 99 papers reporting on 87 unique studies with 51,904 participants from 32 LMICs met the inclusion criteria. Study designs included cross-sectional (n = 89), cohort (n = 6) and experimental (n = 4). Overall, 5 scored as high quality, 79 studies scored as moderate and 15 scored as weak quality. Twenty-nine papers reporting on 22 unique studies used validated alcohol use tools including AUDIT, CAGE and WHO CIDI. The pooled prevalence of any hazardous/harmful/dependent alcohol use was 41% (95% CI: 31–51%), and of daily alcohol use was 26% (95% CI: 17–36%). There was variation in harmful alcohol use by global region (Sub-Saharan Africa: 38%; South Asia/Central Asia/ East Asia and Pacific: 47% and Latin America and the Caribbean:44%). Harmful alcohol use was significantly associated with inconsistent condom use (pooled unadjusted RR: 1.65; 95% CI: 1.01–2.67), STIs (pooled unadjusted OR: 1.29; 95% CI 1.15–1.46); and other drug use (pooled unadjusted OR of 2.44; 95% CI 1.24–4.80), but not with HIV, violence or mental health problems. We found a high prevalence of problem alcohol use and daily alcohol use among FSWs in LMICs. Harmful drinking was associated with important HIV risk factors such as inconsistent condom use, STIs and other drug use. Major limitations included heterogeneity in tools and cut-off scores to measure alcohol use and other common risk factors, and a paucity of longitudinal studies. There is an urgent need for tailored interventions for FSWs in LMICs that address alcohol use as well as the associated sex work risk environment.

## Introduction

Harmful alcohol use is a major public health concern globally, contributing to 3 million deaths every year [1] and increasing the risk of many non-communicable diseases such as liver disease, infectious diseases such as human immunodeficiency virus (HIV) [2], mental health problems such as depression, and harm from external causes such as injuries and violence [3]. Among people aged 15–49 it is the leading risk factor for premature mortality and disability [1, 4]. In recent years alcohol use has been increasing in many LMICs [3]. Increased alcohol regulations to combat alcohol-related harms in high income countries [3] has led the alcohol industry to seek new sources for profit amongst populations with previously lower levels of alcohol consumption [5, 6]. This has concerning implications for both direct and indirect alcohol-related harms [5, 7]. Alcohol use patterns and alcohol-related harms vary globally and by gender and socio-economic status. In a multi-country European study, lower educated men were more at risk of heavy episodic drinking whilst amongst women, higher education was associated with heavy drinking [8]. In sub-Saharan Africa modelling has shown that high-income earners have the highest prevalence of any alcohol use, while low-income earners consume more alcohol per person, and have a higher burden of alcohol-related harm [9]. The need to address alcohol use is included in the Sustainable Development Goals [10], highlighting increased awareness of the burden of alcohol and related harms in LMICs, particularly among high risk groups.

Sex work—defined as the receipt of money or goods in exchange for sexual services—is criminalised in most parts of the world [11, 12]. FSWs face unique occupational risks including sexual and physical violence from clients, and high levels of HIV and other sexually transmitted infections (STI) as well as structural inequalities including police arrest, discrimination, poverty, and gender inequality. In addition, alcohol is widely available in the sex work industry [13, 14] with sex work commonly taking place in venues such as bars with high alcohol availability and women reporting alcohol use to cope with the daily challenges of sex work [14–17]. These factors may predispose FSWs to increased risks of harmful alcohol use. Socio-cultural and economic factors have an effect on the structural and occupational risks associated with sex work [12, 16]; for example differing levels of sex work criminalisation and access to sexual health services mean that alcohol use and associated risks are likely to differ for sex workers in LMICs compared to those in high-income countries. In 2010, Li et al. conducted an integrative review exploring the use of alcohol among FSWs globally, and reported that 81.2–100% of FSWs had ever used alcohol and 73.3–74.8% had used alcohol in the past month [14]. However, the review highlighted several limitations in the currently available literature including the lack of use of validated measurement tools and no meta-analysis was carried out. Additionally, the review did not disaggregate data by low income vs. high income settings. Many studies used very general terms for alcohol use with no further details in quantity, frequency or specified time period. Overall, they reported that problem drinking was under-investigated with no studies using validated tools such as the Alcohol Use Disorders Identification Test (AUDIT) tool [17] to quantify hazardous or harmful use.

Definitions of problem alcohol use vary in the literature, and encompass a spectrum from hazardous to harmful to dependent alcohol use that corresponds to the AUDIT tool (the most commonly used alcohol measurement tool), and other alcohol use tools such as CAGE (Cutting down, Annoyance, Guilty feelings and an Eye-opener) [18] and CIDI (Composite International Diagnostic Interview) [19]. Hazardous alcohol use (AUDIT ≥7/≥8, AUDIT-C ≥3, CAGE ≥2) is usually defined as a pattern of alcohol consumption that increases someone’s risk for physical and/or psychological harm. Harmful alcohol use (AUDIT score 16–19) is defined as a pattern of alcohol consumption that is causing mental or physical health problems, while alcohol dependence (AUDIT score > 20) is defined as craving, tolerance, and preoccupation with alcohol alongside continued drinking in spite of harmful consequences [20, 21]. Since Li’s review there have been a range of studies among FSWs in LMICs reporting on alcohol use with an increase in the use of validated tools such as AUDIT. A recent systematic review of alcohol use among occupational groups at high risk of HIV in sub-Saharan Africa [22] reported that the pooled prevalence of alcohol misuse among sex workers was 45.3% (IQR 25.1–52.0%, 12 studies) but this was not specific to FSWs and was focussed only on sub-Saharan Africa. In addition to harmful alcohol use, there is substantial evidence demonstrating that FSWs in LMICs experience multiple stressors including poverty, lack of education, gender-based violence, mental health problems, drug use, high rates of HIV and STIs and stigma and discrimination [12, 16, 23, 24]. Many of the interconnected health and social issues faced by FSWs can be considered through a syndemics framework. Syndemics are defined by the clustering of two or more diseases in a population, the biological, social and psychological interaction between those diseases and the wider political and socio-economic context that drive the risk of these diseases [25, 26]. As a result, it is important to consider not just the burden of alcohol use, but the key associated risk factors. The review from Li et. al and several other studies among FSWs have reported common associations between alcohol use and poor physical health [14], illicit drug use [27, 28], mental health problems, violence [27], low condom use [27, 29–32], and HIV/STIs [27, 33] but there has been no synthesis of this evidence to date. The need for this systematic review was identified as part of the Maisha Fiti Study, a mixed-method, longitudinal study with FSWs in Nairobi examining associations between violence, mental health, harmful alcohol and other drug use, biological changes to the immune system and HIV/STI prevalence and risk factors. Recently published data from baseline, found that a third (29.9%; 95%CI 27.0–32.6%) of FSWs reported harmful (moderate/high risk) alcohol use, according to the WHO ASSIST tool [34]. Findings from Maisha Fiti identified a gap in the literature on the burden of alcohol use among FSWs, its associated risks and the need for evidence-based interventions.

High quality evidence on the burden of alcohol use among FSWs is key at the global and country level to guide policymaking and develop interventions tailored to address the syndemic health and social challenges faced by FSWs. This systematic review aims to provide an estimate of the prevalence of harmful alcohol use among FSWs in LMICs, and to examine associations with common health and social concerns among this group such as violence, condom use and HIV/STIs to inform intervention development.

## Methods

### Search strategy and selection criteria

**Fig 1:**
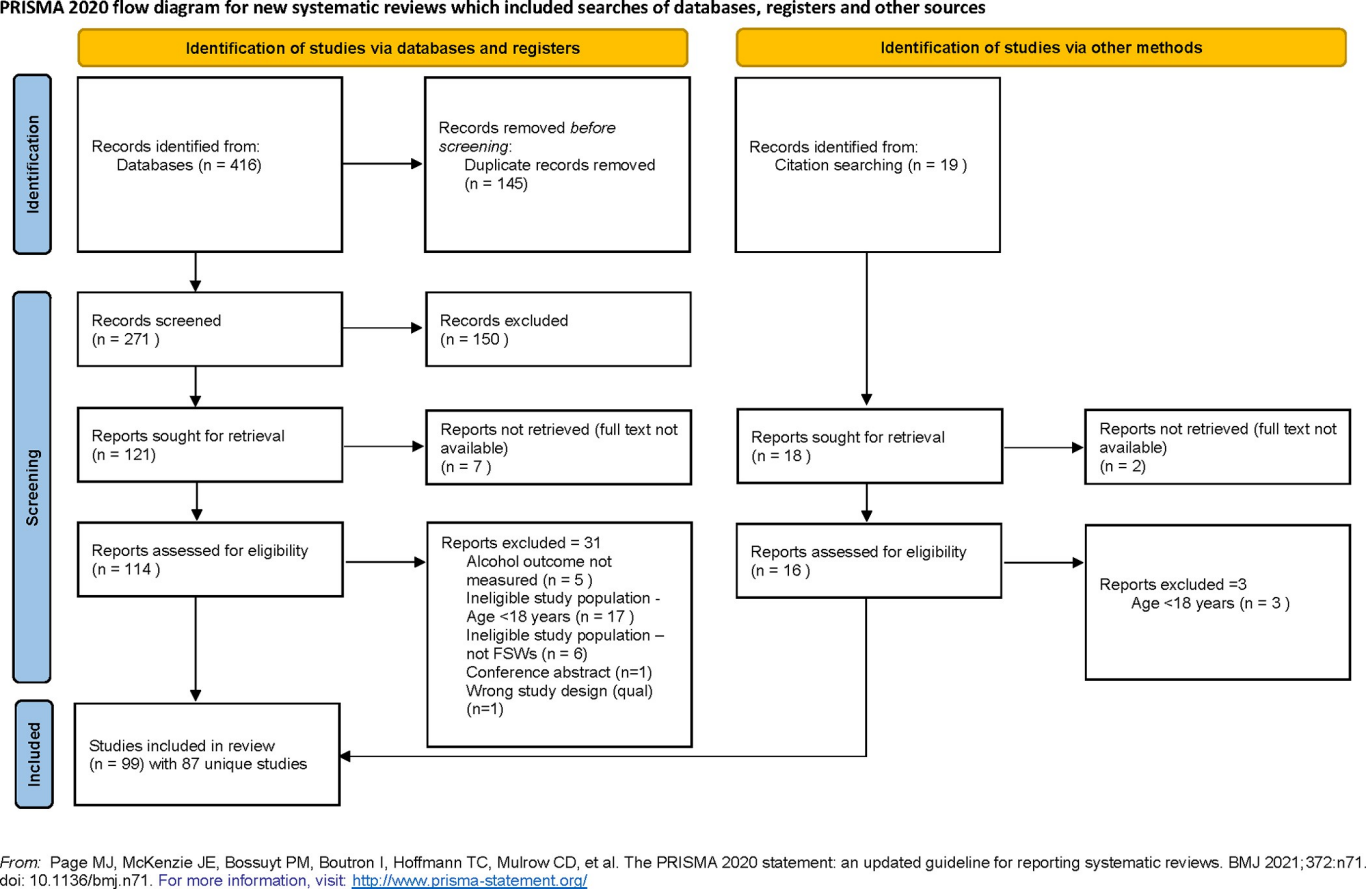
**Study selection.** PRISMA 2020 flow diagram.

The review protocol is registered with PROSPERO, number CRD42021237438 (https://www.crd.york.ac.uk/prospero/). We used the Preferred Reporting Items for Systematic reviews and Meta-Analysis (PRISMA) guidelines (Fig 1). We searched three electronic peer-reviewed literature databases include Ovid (EMBASE, PsycINFO, Global Health, Ovid MEDLINE), PubMed and Web of Science from first record until 24.02.2021. The following search terms were used: AUD OR "alcohol use disorder*" OR alcoholism OR alcohol addict* OR alcohol abuse OR alcohol dependence OR alcohol misuse OR heavy drinking OR binge drink*; “sex work*” OR “female sex work*” OR “FSW*” OR “prostitut*” OR “female prostitut*” OR “sex trad*” OR “transact* sex” OR “commercial sex” OR “sex-trade worker*”; “developing countr*” OR “less developed countr*” OR “under developed countr*” OR “underserved countr*” OR “deprived countr*” OR “poor countr*” OR “transition* countr*” OR “names of countries which fit world bank criteria for LMIC”. (see S1 Appendix for search strategy). We additionally utilised citation list searching to source other eligible studies, as per PRISMA guidelines [35].

### Inclusion criteria

- This review included studies that reported any measure of prevalence or incidence of alcohol use or associations with alcohol use on the basis of a clinical interview, self-reported or clinical examinations among FSWs even if sex workers were not the main focus of the study.
- Studies were included from countries defined as low or middle income, in accordance with the World Bank income groups 2019 [36].
- Eligible studies had to be peer-reviewed.
- Eligible studies included females aged 18 or older who were actively engaged in sex work.
- The following study designs were included: cross-sectional survey, case–control study, cohort study, case series analysis, or experimental study with baseline measures for alcohol use.

- Studies were limited to English language.

### Exclusion criteria

- FSWs who identified as trans sex workers were excluded. This is because transgender sex workers risks and experiences of sex work are considered to be significantly different.
- We excluded studies that used qualitative methods only, were review papers, conference abstracts or non-peer reviewed publications.
- Studies not disaggregating data by alcohol for example referring to ‘alcohol/drug use’ were excluded from this review.
- Studies focused on women engaged in transactional sex only, were ineligible for review, as this practice, and its implications on health, is distinct from sex work [37].

Two reviewers (OK and AB) independently screened all publications in Covidence (https://www.covidence.org/reviewers/) according to the inclusion/exclusion criteria. If Covidence reported a conflict between reviewers on whether a study should be included, the abstract was reviewed, discussed and a final decision reached. Once abstracts were screened, the authors reviewed the full text for final eligibility check.

### Quality assessment

Study quality was assessed by two authors (OK and AB) using the Center for Evidence-Based Management (CEBMa) Critical Appraisal for Cross-Sectional Surveys Tool. Assessment criteria included questions on study design, selection bias, statistical power, validity and reliability of measurement tools, statistical significance and confounding (see S2 Appendix for full details of CEBMa tool). The last item on CEBMa was removed (Item 12: “Can the results be applied to your organisation?”) as it was not relevant to this review. OK and AB each scored half the studies–authors compared 10% of the results of scoring and discussed disagreements in scoring to ensure uniformity in the quality assessment process. Each study was rated based on 11 items, and an overall score was calculated. Studies scoring ≥8 out of 11 points were considered high quality, between 5 and 7 were rated moderate quality, and ≤4 were scored as weak quality. Scoring was based on cut offs used in a previous systematic review among FSWs [16]. A breakdown of individual scores and differences in author scoring are shown in Tables 1 and 2, S3 Appendix. Studies scoring as weak quality were not included in the meta-analyses.

**Table 1:** Study characteristics.

Author, year & study design	Country	Sample	Sampling Procedure	Outcome(s) of interest		Events	Sample size	Event rate (%)	Method of assessing outcome(s)	Research quality
SUB-SAHARAN AFRICA										
Bazzi (2019) *Cross sectional*	Kenya	FSWs	Outreach and targeted sampling + snowball sampling and purposive sampling	Alcohol use in the past month		45	45	100.0%	Semi-structured interviews	Low (3)
				Always/often drunk when using alcohol		25	45	56.0%		
Bitty-Anderson (2019)^i^ *Cross sectional*	Togo	FSWs	Time-location sampling	Moderate drinking		275	937	29.4%	AUDIT score 1-6	High (9)
				Hazardous consumption		344	937	36.7%	AUDIT score > 7	
				Binge drinking		406	937	43.4%	Consumption of six or more alcohol drinks at least once per month in one occasion (AUDIT item nr 3).	
Tchankoni (2020)^i^ *Cross sectional*	Togo	FSWs	Venue-based sampling	Moderate drinking		197	952	20.7%	AUDIT-C score 1-3	Moderate (7)
				Hazardous consumption		432	952	45.5%	AUDIT-C using cut off >4.	
Bukenya (2013) *Cross sectional*	Uganda	FSWs	Targeted and snowball sampling	Alcohol use					Structured face-to-face interviews	Moderate (5)
				*Less than once a week*		60	905	6.6%		
				*At least once a week*		421	905	46.5%		
				*Daily*		235	905	26.0%		
Bukenya (2019) *Cross sectional*	Uganda	FSWs	Convenience sampling	Hazardous alcohol use		462	819	56.4%	AUDIT Score ≥ 7	High (9)
Chersich (2007) *Cross sectional*	Kenya	FSWs	Snowball sampling	Drink but do not binge drink		312	719	43.3%	Structured questionnaire Based on WHO definitions of alcohol Never drunk alcohol non-binge drinkers = lifetime use of alcohol but of <five drinks on any occasion in the preceding month Binge drinkers (>five drinks on > one occasion in the previous month	Moderate (5)
				Binge drink		230	719	32%		
				Ever drunk alcohol		542	719	75.4%		
				*Current drinking frequency;*						
				Secondary abstinence		82	542	15.1%		
				One to three times a month		91	542	16.8%		
				One to two times a week		207	542	38.2%		
				Almost every day or every day		162	542	29.9%		
Chersich (2014) *Cohort*	Kenya	HIV-negative FSWs	Snowball sampling	Alcohol abstinence		144	399	36.1%	Lifetime abstinence or no alcohol use in the past 12 months	Moderate (7)
				Low-risk drinking		148	399	37.1%	AUDIT score 1-7	
				Hazardous drinking		69	399	17.3%	AUDIT score 8-15	
				Harmful drinking		38	399	9.5%	AUDIT score > 16	
Coetzee (2018) *Cross sectional*	South Africa	FSWs	Respondent driven sampling	Problem drinking		348	508	81.5%	AUDIT score of ≥3	High (9)
				Frequent and severe binge drinking		278	508	54.7%	Adapted AUDIT score with a cut-off score of ≥6 (A new variable showing severe versus less severe/no binge drinking was created using the 3 original AUDIT-C items and the new volume variable.)	
Fawole (2014) *Cross sectional*	Nigeria	Brothel-based FSWs	Simple random sampling	Alcohol intake		272	305	17.3%	Questionnaire	High (8)
Gezie (2015) *Cohort study*	Ethiopia	FSWs	Random sampling	Problem drinking		115	474	24.26%	CAGE using cut off > 1	High (9)
Goldenberg (2016) *Cross sectional*	Uganda	FSWs	Outreach	Worked under the influence of alcohol/drugs in the previous 6 months		256	400	64.0%	Questionnaire	Moderate (6)
Kiene (2019) *Cross sectional*	Uganda	CSWs	Snowball sampling	Hazardous drinking and alcohol problems		13	75	17.3%	AUDIT using cut-off > 7	Moderate (6)
				Hazardous drinking		4	75	4%	AUDIT using cut-off > 3 for the first three items of the AUDIT	
				Alcohol problems		3	75	3%	AUDIT using cut-off > 3 for the final seven items of the AUDIT	
Lancaster (2016)^ii^ *Cross sectional*	Malawi	HIV-infected FSWs	Venue-based sampling	Alcohol use prior to last vaginal sex with client		41	138	30%	Behavioural survey	Moderate (6)
				Hazardous drinking		39	138	28.3%	AUDIT score 7-15	
				Harmful drinking		17	138	28.3%	AUDIT score 16-19	
				Alcohol dependence		20	138	14.5%	AUDIT score > 20	
Lancaster (2017)^ii^ *Cross sectional*	Malawi	HIV-infected FSWs	Venue-based sampling	Hazardous drinking		29	96	30%	AUDIT score 7-15	Moderate (6)
				Harmful drinking		10	96	10%	AUDIT score 16-19	
				Alcohol dependent		11	96	12%	AUDIT score > 20	
Leddy (2018) *Cross sectional*	Tanzania	FSWs	Venue-based time location sampling	Frequent intoxication during sex work in the past 30 days		207	496	42.0%	Survey	Moderate (5)
				Drink one or more drinks on a typical day of work		408	496	97.0%		
Nouaman (2015) *Cross sectional*	Côte d’Ivoire	FSWs	Convenience sampling	Moderate alcohol user		84	249	33.7%	AUDIT score <8	Moderate (7)
				Hazardous alcohol user		49	249	19.7%	AUDIT score ≥8	
Ochonye (2019) *Cross sectional*	Nigeria	FSWs	Snowball sampling	Drank alcohol in the last 4 weeks					Semi-structured interviewer-administered questionnaire	Moderate (7)
				*Everyday*		53	188	28.2%		
				*At least once a week*		31	188	17.0%		
				*Occasionally*		46	188	25.3%		
Odukoya (2013) *Cross sectional*	Nigeria	FSWs	Venue-based sampling	Alcohol use					Pretested structured questionnaire	Moderate (7)
				*Current alcohol user*		219	323	67.8%		
				*Ex-alcohol user*		24	323	7.4%		
				Most recent drink						
				*Less than a week ago*		196	219	89.5%		
				*A week to a month ago*		14	219	6.4%		
				*More than a month ago*		9	219	4.1%		
				Amount of alcohol consumed per week in standard units						
				*1-50*		88	219	40.2%		
				*51-100*		63	219	28.8%		
				*101-150*		35	219	16%		
				*151-200*		5	219	2.2%		
				*Above 200*		28	219	12.8%		
				Age at first drink						
				*<18 years*		55	243	22.6%		
				*>18 years*		188	243	77.4%		
				Level of drinking						
				*Within 14 units of alcohol per week*		24	219	11.0%		
				*Above 14 units of alcohol per week*		195	219	89.0%		
Parcesepe (2016)^iii^ *Randomised control trial*	Kenya	FSWs- substance using	Non-probabilistic sampling	Hazardous drinking		528	818	64.6%	AUDIT score 7-15	Moderate (7)
				Harmful drinking		290	818	35.5%	AUDIT score 16-19	
L´Engle (2014)^iii^ *Randomised control trial*	Kenya	FSWs- substance using	Non-probabilistic sampling	Hazardous drinking		529	818	65%	AUDIT score 7-15	Moderate (7)
				Harmful drinking		290	818	35.5%	AUDIT score 16-19	
Richter (2013) *Cross sectional*	South Africa	FSWs	Non-probabilistic sampling	Binge drinking;					Binge drinking defined as having five or more alcohol drinks on one occasion.	Moderate (5)
				*Daily*		284	1566	18.1%		
				*Weekly*		408	1566	26.1%		
Wechsberg (2005)^iv^ *Cross sectional*	South Africa	Substance-using FSWs	Non-probabilistic sampling	Alcohol use by age 17		47	93	51%	Self-reported; items not described in detail.	Moderate (6)
				Alcohol use in the past 30 days;						
				*Daily*		17	93	18%		
				*At least twice a week*		22	93	24%		
Wechsberg (2006)^iv^ *Cross sectional*	South Africa	FSWs who self-report cocaine use or have a positive urine test for cocaine	Non-probabilistic sampling	Alcohol use by age 17		47	93	51%	Self-reported; items not described in detail.	Moderate (6)
				Alcohol use in past 30 days:						
				*Daily*		17	93	18%		
				*At least twice a week*		22	93	24%		
Wechsberg (2011) *Randomised clinical trial*	South Africa	FSWs who use substances	Non-probabilistic sampling	Days of drinking in past 30 days		13.4 days (s.d. 9.8)	550	-	Self-reported use based on questionnaire	Moderate (7)
				Days drunk in past 30 days		10.5 days (s.d. 9.2	550	-	Self-reported use based on questionnaire	
				On drinking day, how many drinks on average, in past 30 days		9.2 days (s.d. 11.0	550	-	Self-reported use based on questionnaire	
Wechsberg (2008) *Cross sectional*	South Africa	FSWs who use substances	Targeted sampling	Alcohol prior to sex		91	163	55.8%	Questionnaire. Item not described in detail.	Moderate (5)
Wechsberg (2009) *Cross sectional*	South Africa	FSWs who use substances	Targeted sampling	Lifetime alcohol use		335	335	100%	Questionnaire based on DSM-IV	Moderate (6)
				Lifetime alcohol use disorder:						
				Abuse		270	335	80.6%		
				Dependence		232	335	69.3%		
				Abuse/dependence		287	335	85.7%		
				Past year alcohol use disorder:						
				Abuse		252	335	75.2%		
				Dependence		215	335	64.2%		
				Abuse or dependence		274	335	81.8%		
Weiss (2016) *Cohort*	Uganda	FSWs	Non-probabilistic sampling	Problem drinking		572	1027	56.0%	CAGE using cut-off > 2	Moderate (6)
Wilson (2016)^v^ *Cross sectional*	Kenya	HIV-positive FSWs	Non-probabilistic sampling	Alcohol use problems						Moderate (6)
				*Minimal*		103	357	28.9%	AUDIT score 1-6	
				*Moderate*		57	357	15.9%	AUDIT score 7-15	
				*Severe/possible AUD*		14	357	3.9%	AUDIT score 16 or higher	
White (2016)^v^ *Cohort*	Kenya	HIV-positive FSWs	Non-probabilistic sampling	*Low risk*		116	405	28.6%	AUDIT score 1-6	Moderate (7)
				*Hazardous or harmful*		88	405	21.7%	AUDIT score 7-40	
Yadav (2005) *Cross sectional*	Kenya	HIV-negative FSWs	Random sampling	Daily alcohol use		222	466	53.4%	Behavioral questionnaire	High (8)
Fearon (2019) *Cross sectional*	Zimbabwe	HIV-negative FSWs	Respondent-driven sampling	Alcohol consumption over the past 12 month					Behavioral questionnaire	Moderate (7)
				*Never*		262		42.9%		
				*Once a month or less*		44	611	7.2%		
				*2-4 times/month*		77	611	12.6%		
				*2-3 times/week*		112		18.3%		
				*4 or more times a week*		115	611	18.8%		
				Had more than 6 alcoholic drinks in one night during last 12 months						
				*Never – no alcohol last 12 months*		262	611	42.9%		
				*Never – drank alcohol but no occasions of more than 6 drinks*		169	611	27.7%		
				*Yes at least one occasion*		178	611	29.1%		
MIDDLE EAST AND NORTH AFRICA										
Kabbash (2012) *Cross sectional*	Egypt	FSWs	Random sampling	Alcohol intake in the last month:					Questionnaire	Moderate (5)
				*Daily*		30	431	7.0%		
				*At least once weekly*		70	431	16.2%		
				*2-3 times monthly*		72	431	16.7%		
Karamouzian (2017) *Cross sectional*	Iran	FSWs	Facility-based sampling	Alcohol use ever		466	451	54.5%	Survey	Moderate (5)
				Alcohol use before sex ever		246	520	4.7%		
SOUTH ASIA										
Todd (2010) *Cross sectional*	Afghanistan	FSWs	Venue-based sampling	Alcohol use		26	520	4.7%	Questionnaire	Moderate (5)
				Consuming 3 drinks or less each week		24	26	93.3%		
				Using alcohol or drugs with clients		27	51	53.9%		
Barua (2012) *Cross sectional*	India	FSWs	Respondent driven sampling	Consumption of alcohol		304	426	71.4%	Questionnaire; items not described in more detail.	Moderate (5)
Bowen (2011) *Cross sectional*	India	FSWs	Convenience and snowball sampling	Alcohol use around time of first sex work		126	220	57.3%	Cross-sectional survey	Low (4)
Devine (2010) *Cross sectional*	India	FSWs	Outreach sampling	Regular AOD use at the time of first sex-work		108	186	58.1%	Questionnaire	Moderate (6)
				Alcohol use around the time of first sex-work		101	186	54.3%		
Heylen (2019) *Cross sectional*	India	FSWs	Non-probability sampling, including referrals from NGOs, brokers or other FSWs	Alcohol use frequency	*<1 time per week*	99	589	16.8%	“On average, how often do you have a drink?”	Moderate (5)
					*1-2 days per week*	238	589	40.4%		
					*3-4 day per week*	100	589	17.0%		
					*5-6 days per week*	9	589	1.5%		
					*Every day*	46	589	7.8%		
Iaisuklang (2017) *Cross sectional*	India	FCSWs	Purposive Sampling	Intake of alcohol Alcohol dependence		79 8	100 100	79.0% 8.0%	Sociodemographic data sheet	Low (3)
Pandiyan (2012) *Cross sectional*	India	FCSWs	Recruitment from hospital	Alcohol use		100	100	100.0%	Questionnaire	Low (2)
Patel (2015) *Cross sectional*	India	FSWs	Conventional cluster sampling and time location cluster sampling	Alcohol use past 30 days		948	1986	47.7%	Questionnaire; item not described in further detail	Moderate (7)
Sagtani (2013) *Cross sectional*	Nepal	FSWs	Snowball sampling	Previous history of sexual intercourse under influence of alcohol		96	200	45.7%	Semi-structured questionnaire. Items not described in detail.	Moderate (6)
Samet (2010) *Cross sectional*	India	HIV-infected FSWs	Purposive sampling	Any alcohol use in the last 30 days		80	211	38%	The number who said they had had any alcohol in the last 30 days.	Moderate (5)
				Heavy alcohol use		67	211	32%	Using cut-off >3 drinks in a day or >7 drinks/week.	
				Alcohol dependence		23	211	11%	CIDI	
Singh (2016) *Cross sectional*	India	FSWs	Convenience sampling	Drinking		33	120	27.5%	Item not described clearly	Moderate (6)
Verma (2010) *Cross sectional*	India	Migrant FSWs	Two-stage random sampling	Alcohol use in the last 1 month	*Any alcohol intake*	2115	3412	62.0%	Survey	High (8)
					*All types of alcohol*	662	3412	19.4%		
					*Alcohol use prior to sex*	1853	3412	53.8%		
EUROPE AND CENTRAL ASIA										
Wirtz (2015) *Cross sectional*	Russia	FSWs	Respondent driven sampling	Alcohol use while selling sex last 6 months		523	754	69.4%	Questionnaire; item not described in further detail	Moderate (5)
Davis (2017) *Cross sectional*	Kazakhstan	HIV-positive FSWs	Recruitment through referrals	Hazardous drinking		23	56	41.1%	AUDIT score >3	Moderate (7)
EAST ASIA and PACIFIC										
Nemoto (2013) *Cross sectional*	Thailand	FSWs	Purposive sampling	Alcohol use in the past 12 months		192	205	93.7%	Questionnaire with open-ended questions; items not described more clearly.	Moderate (5)
				Having sex with customers under the influence of alcohol in the past 6 months		165	205	80.4%		
				Having sex with primary partners under the influence of alcohol in the past 6 months		78	121	64.5%		
Chen (2013)^vi^ *Cross sectional*	China	FSWs	Convenience sampling	Probable drinking problem		343	686	50%	AUDIT using cut-off >8	Moderate (7)
				Probably alcohol dependence		182	686	27%	AUDIT using cut-off >13	
				Risk drinking		217	686	32%	AUDIT score 8-15	
				Heavy drinking		78	686	11.3%	AUDIT score 16-19	
				Hazardous drinking		48	686	7%	AUDIT score 20-40	
Chen (2015)^vi^ *Cross sectional*	China	FSWs	Convenience sampling	Risk drinking		322	1022	31.5%	AUDIT score 8-15	Moderate (7)
				Heavy drinking		119	1022	11.6%	AUDIT score 16-19	
				Hazardous drinking		88	1022	8.6%	AUDIT score 20-40	
Couture (2016) *Cross sectional*	Cambodia	FSWs	Convenience sampling	AUDIT-C (last 3 months):	*Abstinence or lower risk use*	15	100	15.0%	AUDIT-C score 0-2	Moderate (6)
					Unhealthy alcohol use	85	100	85.0%	AUDIT-C score 3-12	
				Alcohol use frequency (last 3 months):	Never/less than once a month	60	100	60.0%	questionnaire .	
					2-4 times a month	10	100	10.0%		
					2 or more times a week	30	100	30.0%		
				Number of drinks on a typical day:	1-2 drinks	7	96	7.3%		
					3-4 drinks	13	96	13.5%		
					5 or more drinks	76	96	79.2%		
				Being drunk or intoxicated recently (last 3 months)		81	100	81.0%		
				Heavy episodic drinking (last 3 months)		83	100	83.0%		
				Regular heavy drinking		29	100	29.0%		
Fang (2007)^vii^ *Cross sectional*	China	FSWs	Ethnographic targeted sampling	Sex w/alcohol		133	454	29.2%	Self-administered questionnaire	Low (4)
				Alcohol intoxication last 6 months		149	454	32.8%		
Hong (2007)^vi^ *Cross sectional* ^i^	China	FSWs	Outreach	Alcohol intoxication in past 6 months		149	454	32.8%	“Have you gotten drunk at least once a month in the past 6 months?”	Moderate (5)
Le (2019) *Cross sectional*	Vietnam	FSWs	Time-location sampling	Daily alcohol consumption in past month		223	1861	12.0%	Cross-sectional survey	Moderate (6)
Nemoto (2008) *Cross sectional*	Vietnam	FSWs	Stratified sampling	Alcohol use in the past 12 months		121	136	89.0%	Survey interview; items not described in detail.	Moderate (6)
Liao (2012) *Cross sectional*	China	FSWs	Respondent-driven sampling	Ever drinking alcohol		612	794	77.1%	Questionnaire	Low (4)
Parcesepe (2015) *Cross sectional*	Mongolia	Alcohol-using FSWs	Convenience sampling	Harmful alcohol use		7	222	3.2%	AUDIT score 8-15	Moderate (7)
				Hazardous alcohol use		9	222	4.1%	AUDIT score 16-19	
				Alcohol dependence		206	222	92.8%	AUDIT score >20	
Su (2014) *Cross sectional*	China	FSWs	Venue based sampling	Mean AUDIT score		9.45±6.77	1022	-	AUDIT score	Moderate (6)
Tran (2014) *Cross sectional*	Vietnam	FSWs	Peer-educator and staff referrals	Alcohol use		1663	1999	83.4%	Questionnaire	Low (4)
Urada (2012) *Cross sectional*	Philippines	FSWs	Venue based sampling	Used alcohol		99	142	70%	Questionnaire; item not described in more detail	Moderate (5)
Urada (2014) *Cross sectional*	Philippines	FSWs	Venue-based sampling	Current alcohol use; Daily		71	482	14.7%	“How often do you have beer or drinks containing alcohol?”	Moderate (6)
				Drinks alcohol, not daily		231	482	48%		
				Alcohol use with venue patron ever		165	482	34%	“How often do you drink beer or alcohol with your venue patron?”	
				Alcohol intoxicated during sex ever		159	482	32%	“How often are you drunk when you have sex?”	
Witte (2010) *Cross sectional*	Mongolia	FSWs	Purposive sampling	Alcohol consumption on a typical day		44	48	92%	Items not described in detail.	Low (3)
				1-2 drinks/day		9	48	29%		
				3-4 drinks/day		14	48	29%		
				5-6 drinks/day		13	48	27%		
				7-9 drinks/day		2	48	4%		
				10 or more drinks/day		6	48	13%		
				Being unable to stop drinking once they have started on at least a monthly basis		39	48	81%		
Witte (2011) *Randomized clinical trial*	Mongolia	FSWs (alcohol using FSWs)	Non-probabilistic sampling	AUDIT score >8		229	229	100%	AUDIT	Moderate (6)
Zhang (2017) *Cross sectional*	China	FSWs	Non-probability sampling	Average AUDIT score		8.4	673	-	AUDIT	Low (4)
Zhang (2014)^viii^ *Cross sectional*	China	FSWs	Venue based sampling	Alcohol intoxication		627	968	64.8%	Questionnaire: frequencies of alcohol intoxication (e.g. every day, once 2–3 days, once a week, once 2–3 weeks, never)	Moderate (5)
Zhang (2014)^viii^ *Cross sectional*	China	FSWs	Venue based sampling	Mean audit score (sd)		9.05 (7.36)	1022		AUDIT	Moderate (6)
LATIN AMERICA AND THE CARIBBEAN										
Aguayo (2008) *Cross sectional*	Paraguay	FCSWs	Non-probabilistic sampling	Sexual intercourse under the effects of alcohol	Sometimes	341	723	47.2%	Questionnaire; items not described.	Low (4)
					Always	106	723	14.7%		
Bautista (2006) *Cross sectional*	Argentina	FSWs	Non-probabilistic sampling	Use of alcohol		36	1782	2.0%	Questionnaire; item not described	Low (4)
Bazzi (2015) *Cohort study*	Mexico	FSWs	Targeted and snowball sampling	Hazardous/harmful alcohol drinking in the past 6 months		42	212	20.0%	AUDIT using cut-off <8	Moderate (7)
Caetano (2013) *Cross sectional*	Brazil	FSWs	Respondent-driven sampling	Alcohol use last month	*Every day*	144	395	36.4%	Standardized questionnaire adapted from the FSW module of the Health International Behavioral Surveillance Surveys	Moderate (5)
					*Three times a week*	131	395	33.2%		
					*At least once a month*	49	395	12.4%		
Carrasco (2019) *Cross sectional*	Dominican Republic	HIV-positive FSWs	Non-probabilistic hybrid sampling	Alcohol use		83	223	37.2%	Alcohol use: Study participants were asked about alcohol use in the last 30 days.	Moderate (6)
Chen (2012) *Cross sectional*	Mexico	FSWs	Modified venue-based sampling	Heavy alcohol use	*1-4 drinks on typical drinking day*	30	174	17.2%	Quantitative surveys	Moderate (6)
					*>4 drinks on typical drinking day*	144	174	82.8%		
				Frequency of alcohol use	*Never-1x/week*	58	189	30.7%		
					*Several times/week- daily*	131	189	69.3%		
Conners (2016) *Cross sectional*	Mexico	FSWs	Modified time-location sampling	Use alcohol often or always before sex		117	496	24.0%	Survey	Moderate (7)
Costa Passos (2004) *Cross sectional*	Brazil	FSWs	Venue-based sampling and snowball sampling	Being under the influence of alcohol	Daily/weekly	287	462	62.1%	Questionnaire	Moderate (6)
					Rare/never	175	462	37.9%		
Dal Pogetto (2012) *Cross sectional*	Brazil	FSWs	Non-probabilistic sampling	Use alcohol at work		86	102	84.3%	Interview	Moderate (6)
Damacena (2014) *Cross sectional*	Brazil	FSWs	Respondent-driven sampling	Frequency of alcohol consumption	*Moderate (around once a week or less)*	1160	2523	46.0%	Questionnaire in an Audio Computer-Assisted Self-Interview	Moderate (6)
					*Elevated (several times a week or every day)*	700	2523	27.7%		
de Matos (2017) *Cross sectional*	Brazil	FSWs	Respondent-driven sampling	Alcohol use in the last month	*No/only once a month*	95	293	21.5%	Questionnaire; items not described.	Moderate(7)
					*At least three times a week*	103	293	21.0%		
					*Everyday*	95	293	22.8%		
Devoglio (2017) *Cross sectional*	Brazil	FSWs	Non-probabilistic sampling	Consumes alcohol	74	83	89.2%	Questionnaire	Moderate (7)
Donastorg (2014) *Cross sectional*	Dominican Republic	HIV-positive FSWs	Non-probabilistic hybrid sampling	Alcohol use in last 30 days	*At least once a week*	125	298	35.4%	Socio-behavioral survey	Moderate (5)
					*Less than weekly*	173	298	64.6%		
				Alcohol use before sex *Sometimes/Always*	125	267	46.8%			
Duncan (2010) *Cross sectional*	Jamaica	FSWs	Random sampling	Everyday alcohol use		264	450	58.7%	Questionnaire	Low (3)
Gaines (2013) *Cohort*	Mexico	FSW-IDUs	Outreach	Weekly alcohol consumption		248	567	43.7%	Face-to-face interviews	Low (4)
Goldenberg (2012) *Cross sectional*	Mexico	FSWs	Outreach	Used alcohol before starting sex work		403	624	64.4%	Questionnaire	Moderate (5)
Hooi (2018) *Cross sectional*	Curacao	FSWs	Random sampling	Alcohol use		29	76	39.2%	Survey	Low (4)
Jain (2018) *Cross sectional*	Mexico	FSW-IDUs	Random sampling	Alcohol use before or during sex with clients		296	584	50.8%	Survey	Moderate (5)
				Binge drinking - Five or more alcoholic beverages in one sitting in the past months		271	584	46.5%		
Jain (2020) *Cross sectional*	Mexico	FSWs	Random sampling	Hazardous alcohol consumption in the past year		136	295	46.1%	AUDIT using cut-off >8	Moderate (6)
Kerrigan (2016) *Cross sectional*	Dominican Republic	FSWs	Hybrid sampling; recruitment by FSW peer navigators and referrals	Alcohol use in the last 30 days	*At least once a week*	123	228	53.9%	Interviewer-administered socio-behavioral survey	Moderate (7)
					*Less than once a week*	105	228	46.1%		
				Alcohol use before sex *Sometimes/always*	108	228	47.4%			
Munoz (2006) *Cross sectional*	Venezuela	FCSWs	Time-location sampling	Alcohol consumption;	1 or 2 drinks/month	104	613	17.0%	Questionnaire; items not described.	Lowe (4)
					1 drink/week	188	613	30.7%		
					1 drink/day	56	613	9.1%		
					>1 drink/day	73	613	11.9%		
Munoz (2010) *Cross sectional*	Mexico	FSWs	Recruitment through outreach	Used alcohol in the last month		673	924	73.0%	Baseline survey face-to-face	Moderate (6)
				Used alcohol during or before sex work		546	924	59.0%		
Pando (2006) *Cross sectional*	Argentina	FCSWs	Snowball sampling	Alcohol consumption;	<Once a week	486	625	77.8%	Standardised questionnaire; items not described in detail.	Moderate (6)
					>Once a week	139	625	22.2%		
Persaud (2000) ^i^ *Cross sectional*	Guyana	FCSWs	Non-probabilistic sampling	Always under the influence of alcohol while having sex with their last 10 clients		43	124	34.5%	Questionnaire	Low (3)
Persaud (1999) ^vii^ *Cross sectional*	Guyana	FCSWs	Non-probabilistic sampling	Regular alcohol consumption while looking for clients		119	124	88.0%	Questionnaire	Low (3)
Salazar (2019) *Cross sectional*	Mexico	FSWs	Time-location sampling	First month alcohol use		402	603	66.0%	Questionnaire	Moderate (5)
				First month forced alcohol use		26	650	4.0%		
Semple (2016)^ii^ *Cross sectional*	Mexico	HIV-negative FSWs	Time-location sampling	Hazardous drinking		835	1089	76.7%	AUDIT-C using cut-off >3	Moderate (6)
				Used alcohol before or during sex with client		651	1089	65.0%	“Have you used alcohol before or during sex with client in the past month?”	
Semple (2015)^viii^ *Cross sectional*	Mexico	HIV-negative FSWs	Time-location sampling	Used alcohol before or during sex with client(s) in past month		661	1089	60.7%	Participants were asked how often in the past month they had used alcohol before or during sex with a client. Response categories (never, sometimes, often, always) were recoded yes/no to create two dichotomous variables	Moderate (6)
Semple (2017)^viii^ *Cross sectional*	Mexico	HIV-negative FSWs	Time-location sampling	Hazardous alcohol use		835	1089	76.7%	AUDIT-C using cut-off >3	Moderate (6)
				Used alcohol with client in past month		661	1089	60.7%	Item not described in detail	
Servin (2017) *Cross sectional*	Mexico	FSWs	Time-location sampling	Always used alcohol right before or during sex with clients in the past 30 days		381	603	63.7%	Questionnaire	Moderate (5)
Strathdee (2008) ^iii^ *Cross sectional*	Mexico	FSWs	Venue based sampling	Often/always used alcohol before/during vaginal sex		207	924	22.4%	Questionnaire; items not described in detail	Moderate (6)
Patterson (2006) ^ix^ *Cross sectional*	Mexico	FSWs	Recruitment through health clinics, street outreach and referrals.	Alcohol use in the past month		273	295	93.0%	Interviewer-administered survey	Moderate (5)
Ulibarri (2014)^ix^ *Cross sectional*	Mexico	FSWs	Non-probabilistic sampling	Used alcohol in the past month		673	924	72.8%	Interview; items not described in detail.	Moderate (5)
				Used alcohol before sex with clients		297	924	32.1%		

^i^ Bitty-Anderson (2019) and Tchankoni (2020)–papers report on same study

^ii^ Lancaster (2016) and Lancaster (2017)–papers report on same study

^iii^ L’Engle (2014) and Parcesepe (2016)–papers report on same study

^iv^ Wechsberg (2006) and Wechsberg 2005 –papers report on same study

^v^ Wilson (2016) and White (2016)–papers report on same study

^vi^ Chen (2013) and Chen (2015)–papers report on same study

^vii^ Hong (2007) and Fang (2007)–papers report on same study

^viii^ Zhang (2014) and Zhang (2014)–papers report on same study

^ix^ Persaud (2000) and Persaud (1999)–papers report on same study

**Table 2:** Associations with alcohol use (cross-sectional studies).

Author & Study	Country	Sample	Alcohol use measure	Outcome of interest	Sample size	Odds in the exposed^1^	Odds in the unexposed^2^	Crude Odds Ratio (95% CI)	P-value
**Violence and arrest**									
Chersich (2014)*	Kenya	HIV-negative FSWs	Hazardous/ Harmful drinking (AUDIT score ≥ 8)	Sexual violence (physically forced to have sex)	399	39/68	32/260	4.66 (2.72–7.98)	<0.001
				Physical violence	399	77/30	59/233	10.13 (6.09–16.87)	<0.001
Jain (2020)	Mexico	FSWs	AUDIT using cut-off >8	Lifetime experience of physical abuse or sexual violence perpetrated by a client.	295	58/49	78/110	1.67 (1.04–2.69)	0.04
Semple (2016)	Mexico	FSWs	AUDIT-C using cut-off >3	Forced to have sex with client in past year	1001	118/717	27/139	0.85 (0.54–1.34)	0.5
Semple (2016)	Mexico	FSWs	AUDIT-C using cut-off >3	Ever arrested	1001	176/659	39/127	0.87 (0.59, 1.29)	0.5
**Condom Use**										
Weiss (2016)	Uganda	FSWs	CAGE using cut-off > 2	Inconsistent condom use (time period not specified)	905	218/298	146/243	1.22 (0.93–1.59)	0.2
Chen (2013)	China	FSWs	AUDIT using cut-off >8	Inconsistent condom use with stable partner most recent 3 episodes of sex	983	285/260	182/256	1.54 (1.20–1.99)	0.01
				Inconsistent condom use with casual partner most recent 3 episodes of sex	983	207/338	90/348	2.37 (1.77–3.16)	<0.001
Semple (2016)	Mexico	HIV negative FSWs	AUDIT-C using cut-off >3	Unprotected vaginal and anal sex acts with clients	1001	131/704	28/138	1.04 (0.73–1.49)	0.7
**Other sexual risk behaviours**										
Bukenya (2019)	Uganda	FSWs	Hazardous alcohol use (AUDIT Score ≥ 7)	Unplanned pregnancy	819	319/143	214/143	1.5 (1.12–1.99)	0.007
Semple (2016)	Mexico	FSWs	AUDIT-C using cut-off >3	Total number of clients	1001	430/404	108/58	0.52 (0.37–0.74)	0.002
Chersich (2014)	Kenya	HIV-negative FSWs	Hazardous or harmful drinking (AUDIT score ≥ 8)	using any contraception (condoms/oral contraception, injectable or implant)	399	64/43	189/103	0.81 (0.51–1.28)	0.4
**HIV/STI prevalence**									
Couture (2016)	Cambodia	FSWs	AUDIT-C score 3–12	HIV prevalence	100	4/81	5/10	0.1 (0.03–0.43)	0.002
Nouaman (2015)	Côte d’Ivoire	FSWs	AUDIT score ≥8 (hazardous)	HIV prevalence	249	11/73	38/127	0.50 (0.24–1.05	0.07
Weiss (2016)	Uganda	FSWs	CAGE using cut-off > 2	HIV prevalence	1027	225/347	156/299	1.24 (0.96–1.61)	0.1
				HSV-2	1027	474/98	348/107	1.49 (1.09–2.02)	0.01
				Active syphilis	1027	61/508	42/413	1.18 (0.78–1.79)	0.4
				Bacterial vaginosis	1027	327/245	246/209	1.14 (0.89–1.45)	0.3
				Candida ssp.	1027	71/501	41/414	1.43 (0.95–2.15)	0.08
				Trichomonas vaginalis	1027	95/377	81/374	1.16 (0.84–1.62)	0.4
				Neisseria gonorrhoeae	1026	85/457	49/405	1.54 (1.06–2.24)	0.03
				Chlamydia trachomatis	1026	51/521	41/413	0.99 (0.64–1.52)	0.9
				Mycoplasma genitalium	1025	94/478	54/399	1.45 (1.01–2.08)	0.04
Chersich (2014)	Kenya	HIV-negative FSWs	Hazardous/harmful drinking (AUDIT score ≥ 8)	STI prevalence (syphilis or trichomonas)	399	10/97	14/278	2.05 (0.88–4.76)	0.1
Chen (2013)	China	FSWs	AUDIT using cut-off >8	History of STI	983	46/499	28/410	1.35 (0.83–2.20)	0.2
Jain (2020)	Mexico	FSWs	AUDIT using cut-off >8	Tested positive for chlamydia, gonorrhea, or active syphilis.	295	27/107	27/132	1.33 (0.74, 2.37)	0.3
**Mental Health Problems and drug use**										
Jain (2020)	Mexico	FSWs	AUDIT using cut-off >8	Polydrug use last month (Use of ≥2 illicit drugs (heroin; methamphetamine; cocaine; inhalants; ecstasy; tranquilizers; barbiturates) in the past month)	295	44/92	41/118	1.38 (0.83–2.28)	
				Moderate or severe depression defined as a score ≥20 on the Beck Depression Inventory II (BDI-II).	295	57/79	49/110	1.62 (1.00–2.62)	0.05
Chersich (2014)	Kenya	HIV-negative FSWs	Hazardous or harmful drinking (AUDIT score ≥ 8)	Cannabis in past week	399	17/90	13/279	4.05 (1.90–8.67)	0.0003
Chersich (2014)	Kenya	HIV-negative FSWs	Hazardous or harmful drinking (AUDIT score ≥ 8)	Khat use in past week	399	35/72	39/253	3.15 (1.86–5.34)	<0.0001
Semple (2016)	Mexico	FSWs	AUDIT-C using cut-off >3	Used drugs in past month	1001	99/736	7/159	3.06 (1.39–6.70)	<0.01
Coetzee (2018)	South Africa	FSWs	Adapted AUDIT-C score with cut off of ≥6 (frequent and severe binge drinking)	Depression (20-item CES-D scale)	508	196/81	174/55	0.76 (0.51–1.14)	0.2
				PTSD (PTSD-8)	508	99/179	96/134	0.77 (0.54–1.11)	0.2
Tchankoni (2020)	Togo	FSWs	AUDIT score > 7	Psychological distress (Kessler)	952	82/99	350/421	1.0(0.7–1.4)	1.0

*Cohort study but some associations reported at baseline.

^1^ odds in the exposed (e.g. alcohol and violence/alcohol and no violence).

^2^ odds in the unexposed (e.g. no alcohol and violence/no alcohol and no violence).

### Data extraction and analysis

Data were extracted by two authors (OK and AB) into a structured data extraction document (Table 1) to include data on: author, study design, publication year, country of publication, study design, sample size, alcohol use measure and types, prevalence of alcohol use, and associations with the outcome. Studies that met inclusion criteria were organised by similar findings and reported through a narrative synthesis. Prevalence estimates were calculated from percentages or proportions. Meta-analyses were conducted to estimate global or regional prevalence on studies that scored moderate or high in quality assessment. Through narrative synthesis of available measures (e.g. frequency of alcohol use) and known validated tools (e.g. AUDIT, CAGE) we selected a range of measures of alcohol use and harmful alcohol use for meta-analyses. Where results from a single study were reported in multiple papers, we included all studies in Table 1 but only the original study was reported in the prevalence analyses. The global pooled estimates for the mean prevalence of alcohol use and its associated 95% confidence intervals (95% CI) were calculated in Stata using random effects models. Heterogeneity was measured through the Higgins’ I^2^ statistic, which gives the percentage of total variation across studies in a meta-analysis that is due to heterogeneity rather than chance, ranging from 0–100% [38]. A high I^2^ statistic would indicate greater heterogeneity between studies. Analyses were conducted in STATA 16.1 (Stata Inc., College Station, TX, USA).

For all included studies, any associations with alcohol use were reported in narrative synthesis. Only studies using validated alcohol measurement tools were eligible to have associations data included in meta-analyses. Sub-group analyses examined associations between alcohol use (measured using a validated tool) and common health and social concerns for FSWs including HIV status, STIs, condom use, other drug use, violence experience and mental health.

## Results

### Study characteristics

The initial electronic search yielded 416 results, with 19 additional studies identified through citation searching. After duplicate records were removed, the titles and abstracts of 290 publications (271 from databases, 19 from other sources) were screened for eligibility. Of these, 140 full texts were identified as potentially relevant publications and reviewed for inclusion. Ninety-nine papers reporting on 87 unique studies with 51,904 participants met the inclusion criteria (Fig 1) with year of publication ranging from 1999 to 2020. Studies were based in 32 LMICs: 10 in Sub-Saharan Africa, 2 in the Middle East and North Africa, 3 in South Asia, 2 in Europe and Central Asia, 6 in East Asia and the Pacific and 9 in Latin America and the Caribbean.

#### Study population

The majority of sampling techniques were non-probabilistic, which included convenience (n = 10), purposive (n = 5), snowball (n = 9), venue-based (n = 10), respondent driven sampling (n = 8), outreach (n = 7), targeted (n = 3) and time-location (n = 9). Twenty eight studies reported non-probabilistic sampling with no further detail on sampling methods. Ten studies used probabilistic (simple random sampling) methods (Table 1). Ten studies selected participants based on harmful alcohol or drug use [39–48] and were included in Table 1 but excluded from the meta-analyses (regardless of CEBMa score) to avoid biasing the pooled estimates. Thirteen studies selected participants based on HIV status (n = 6 HIV negative [27, 28, 49–52], n = 8 HIV positive [53–60]) and results were reported with these studies included and excluded in meta-analysis for comparison.

#### Study design and quality

Eighty-nine studies used cross-sectional designs to report on alcohol use, six used a cohort design [27, 33, 47, 55, 61, 62] and four experimental studies reported an alcohol intervention [39, 40, 43, 48]. Five studies scored as high quality, 79 scored as moderate and 15 scored as weak quality (Table 1 and S3 Appendix).

### Patterns of alcohol use

The results on patterns of alcohol use are shown in Table 1. The majority of FSWs drank frequently, though drinking patterns varied with setting and tools used to measure alcohol use. A variety of measures on drinking prevalence and frequency were reported with the majority of studies (75.6%, n = 65 studies) not using a validated tool to assess alcohol use. Overall, 2.0–100.0% of FSWs reported consuming any alcohol (no timeframe) [59, 63–80].

For studies reporting on alcohol use frequency, 12.0–100% used alcohol in the past month [41–43, 50–52, 57, 60, 63, 80–92]; 89.0–93.7% reported using alcohol in the last 12 months [93, 94]; 6.4–77.8.0% used alcohol at least once a week [29, 41, 42, 52, 60, 63, 85, 87–90, 95–102] and 10.0–64.6% used alcohol at least once a month [29, 52, 60, 63, 85, 87, 89, 90, 97].

In terms of drinking patterns, 32.8–81.0% reported being drunk when using alcohol [43, 71, 82, 84, 97, 103–105] and 26.1–54.7% reported binge drinking [52, 63, 83, 95, 106–108].

Alcohol use was common during sex with 32.1–97.0% reporting having used alcohol during sex work [28, 50, 51, 76, 84, 89, 91, 92, 106, 108–115]; 22.4–80.4% reported having any sexual intercourse (with clients/partners or unspecified) under the influence of alcohol [28, 44, 50, 53, 60, 71, 79, 80, 89, 92, 93, 103, 108, 114–119] and 54.3–66.9% used alcohol at the time of first sex work [111, 112, 120, 121].

### Prevalence of alcohol use reported using validated tools

In total, 29 papers from 22 unique studies reported on prevalence of alcohol use using validated tools [27, 28, 32, 33, 39, 40, 46, 48, 51, 53–55, 57, 58, 61, 63, 83, 84, 97, 107, 113, 122–129] (Table 1). Studies were based in thirteen LMICs including 7 in sub-Saharan Africa, 1 in South Asia, 1 in Europe and Central Asia, 4 in East Asia and Pacific and 1 in Latin America. The majority of these studies were cross sectional (n = 25), while two studies were cohort [27, 33] and two were randomised controlled trial [40, 48]. Tools and cut off scores to measure alcohol use disorders varied. In total 26 studies [27, 28, 32, 39, 40, 46, 48, 51, 53–55, 58, 62, 83, 84, 97, 107, 113, 122–129] used the AUDIT tool—three studies reported on mean AUDIT score [127–129], five [28, 51, 58, 97, 123] used the shortened AUDIT-C with score ≥3 as a cut-off and one an adapted AUDIT-C [107]. Of the sixteen studies using the full AUDIT tool, nine used AUDIT cut off ≥7 [29, 39, 40, 53–56, 126, 127] to define hazardous alcohol use and eight used AUDIT cut off ≥8 [27, 32, 46, 48, 62, 113, 122, 126]. Two studies used CAGE [33, 61] with one study using a cut-off of ≥2 and one study using a cut-off of ≥1, and one study used the WHO CIDI tool [57].

### Harmful alcohol use

**Fig 2:**
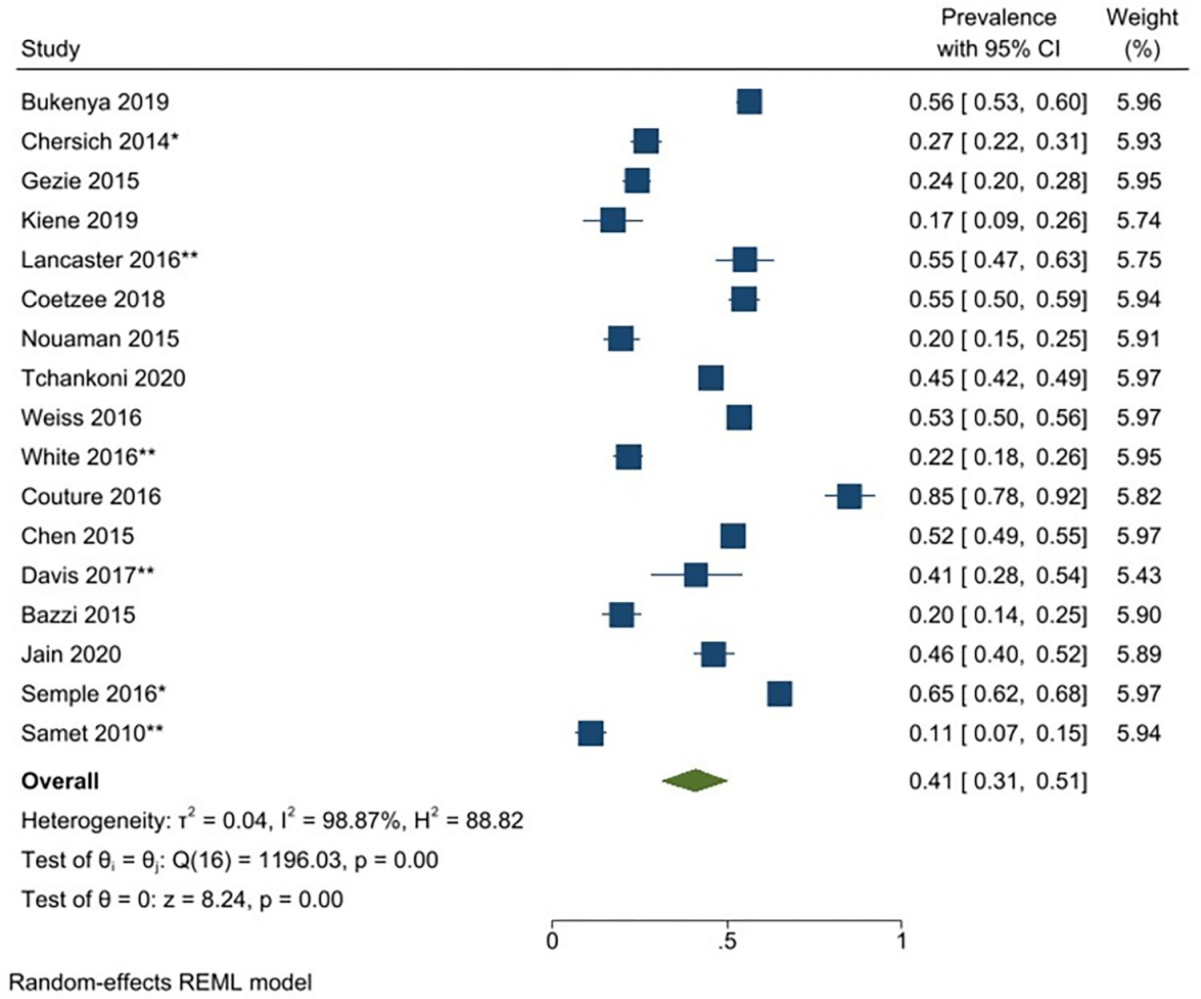
Any hazardous/harmful/dependent alcohol use—pooled prevalence estimates.

**Fig 3:**
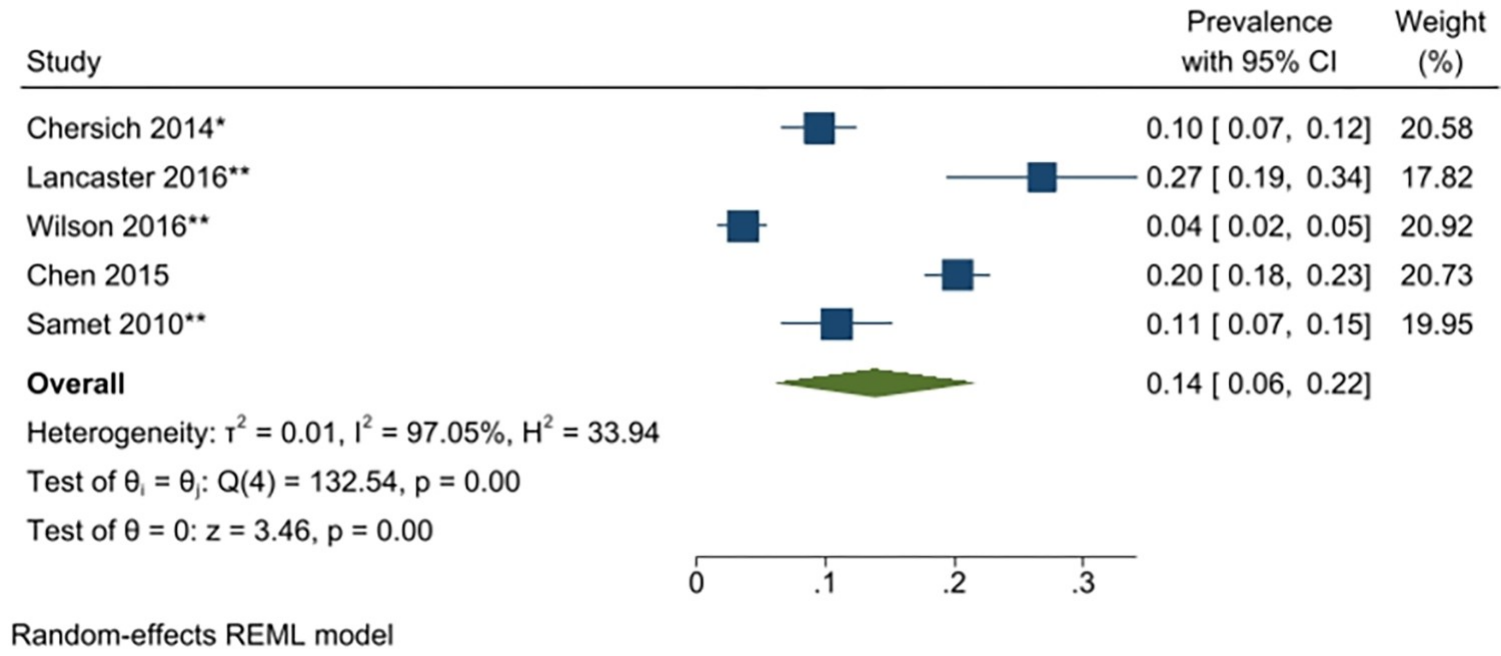
Any harmful/dependent alcohol use—pooled prevalence estimates.

**Fig 4:**
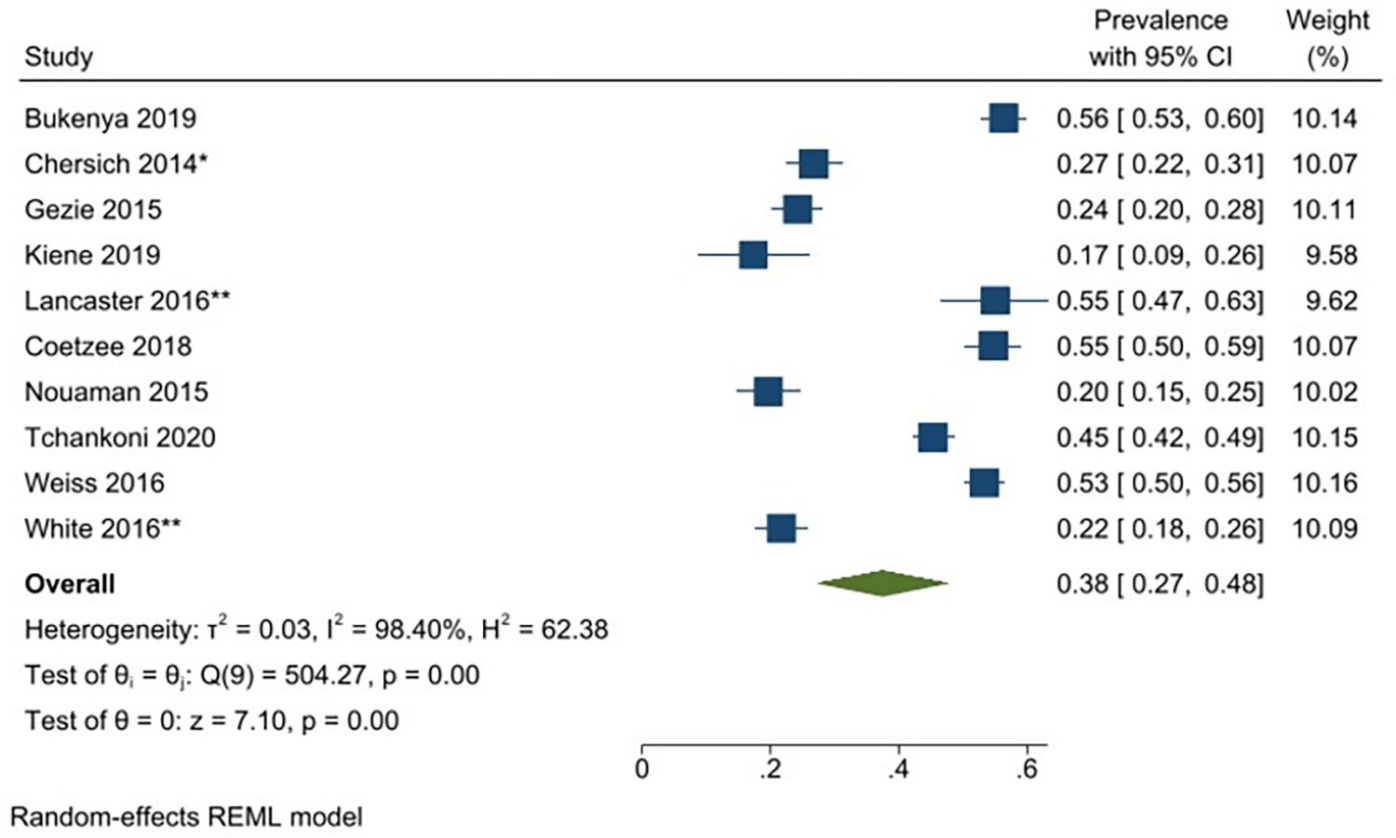
Any hazardous/harmful/dependent alcohol use—pooled prevalence estimates for sub-Saharan Africa.

**Fig 5:**
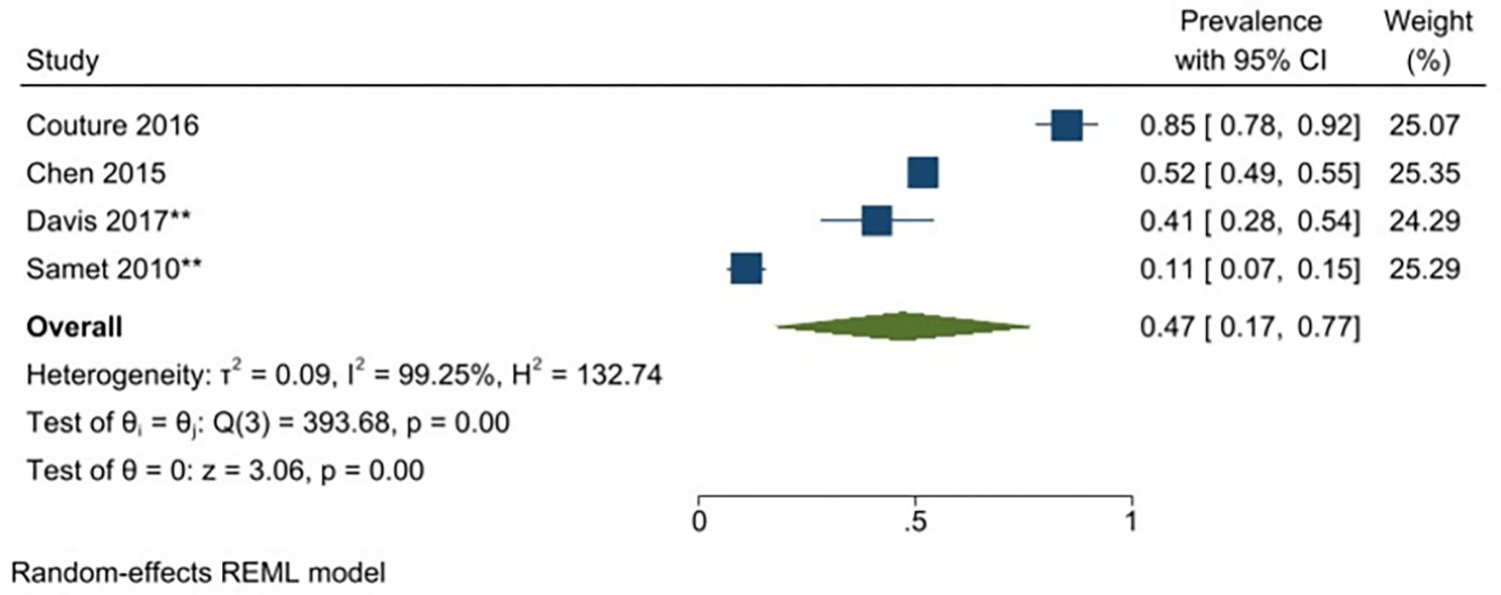
Any hazardous/harmful/dependent alcohol use—pooled prevalence estimates for South Asia/ Central Asia/ East Asia and Pacific.

**Fig 6:**
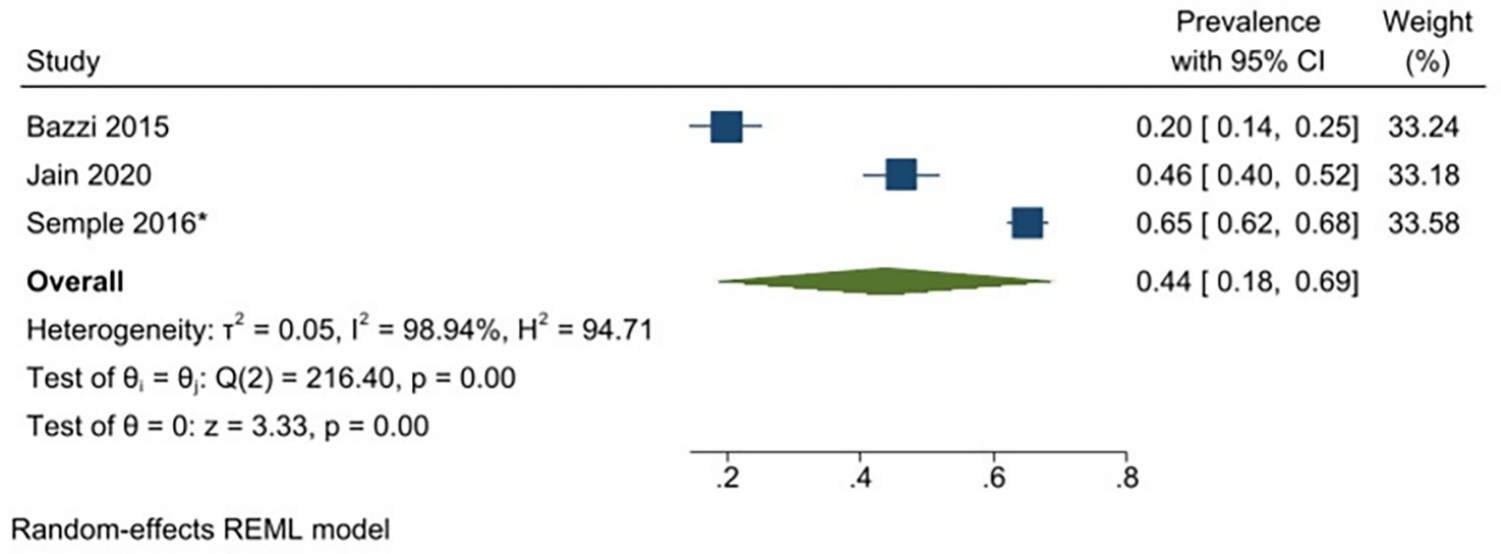
Any hazardous/harmful/dependent alcohol use—pooled prevalence estimates for Latin America and the Caribbean.

A meta-analysis was conducted with seventeen unique studies that used a validated tool (excluding studies that included only substance using FSWs) and estimated the pooled prevalence of any harmful alcohol use to be 41% (31–51%) (*I*^*2*^ = 98.87%) (Fig 2). The same analysis was conducted without the six studies that had selected participants based on HIV-status (as this could potentially bias the findings) and this analysis estimated the pooled prevalence to be similar (43% (95% CI: 31–55%) (*I*^*2*^ = 98.96%)). The pooled prevalence of harmful or dependent alcohol use only (AUDIT ≥16/CIDI tool) was 14% (95% CI: 6–22%)(*I*^*2*^ = 97.05%) (Fig 3). Prevalence estimates were conducted for each of the different regions were there was appropriate data, resulting in an estimated pooled prevalence of harmful alcohol use to be 38% (95% CI: 27–48%)(*I*^*2*^ = 98.40%) in Sub-Saharan Africa (Fig 4); 47% (95% CI: 17–77%) (*I*^*2*^ = 99.62%) in South Asia/ Central Asia/ East Asia and Pacific (Fig 5) and 44% (95% CI: 18–69%) (*I*^*2*^ = 98.94%) in Latin America and the Caribbean (Fig 6). When studies selected based on HIV status were excluded, the results were similar for sub-Saharan Africa (39%; 95% CI: 26–52%; n = 4) but differed for Asia (68%; 95% CI: 36–101%; n = 2) and Latin America (33%; 95% CI: 7–59%; n = 2).

### Daily alcohol use

**Fig 7:**
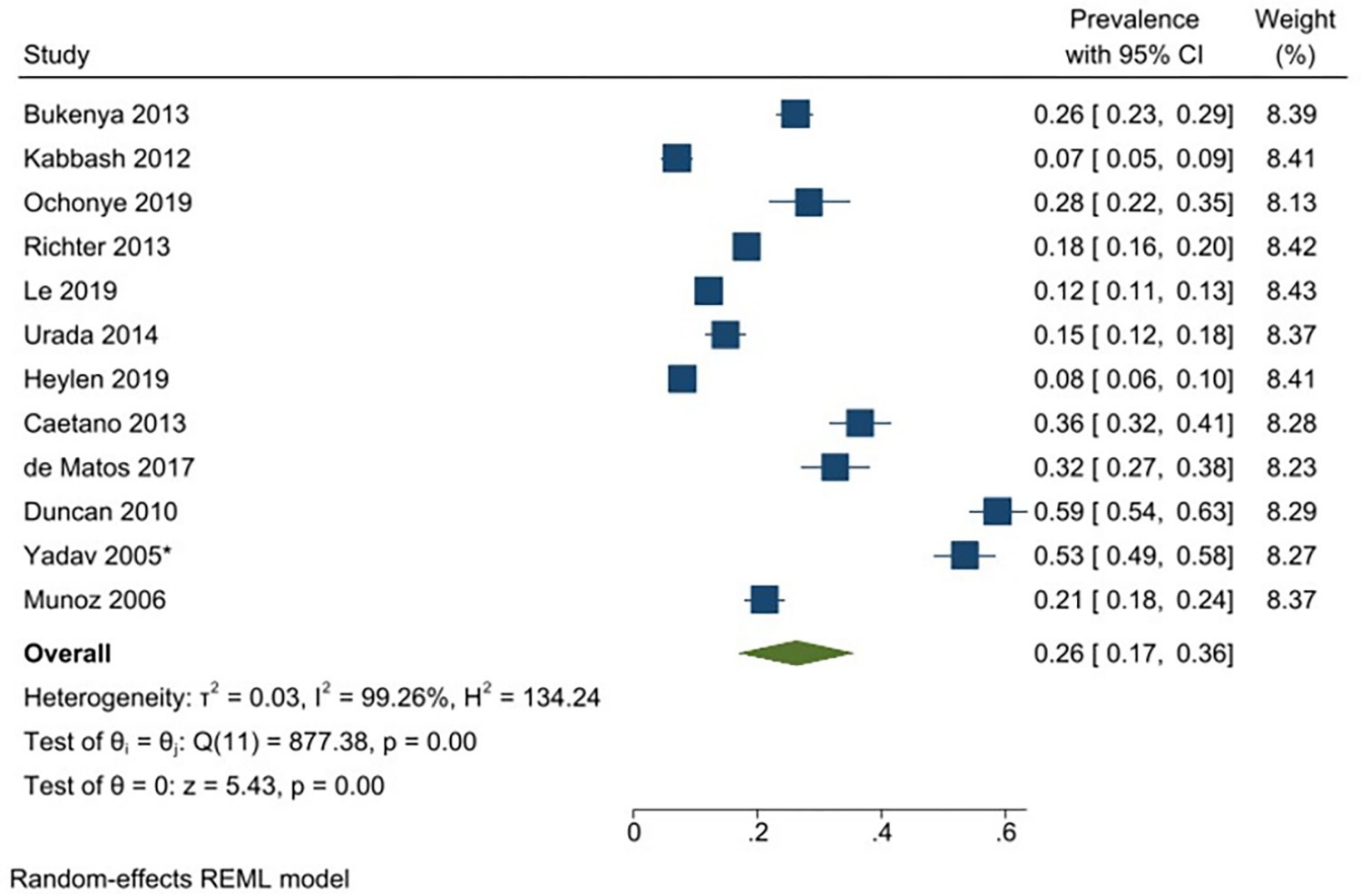
Daily alcohol use—Pooled prevalence estimates.

**Fig 8:**
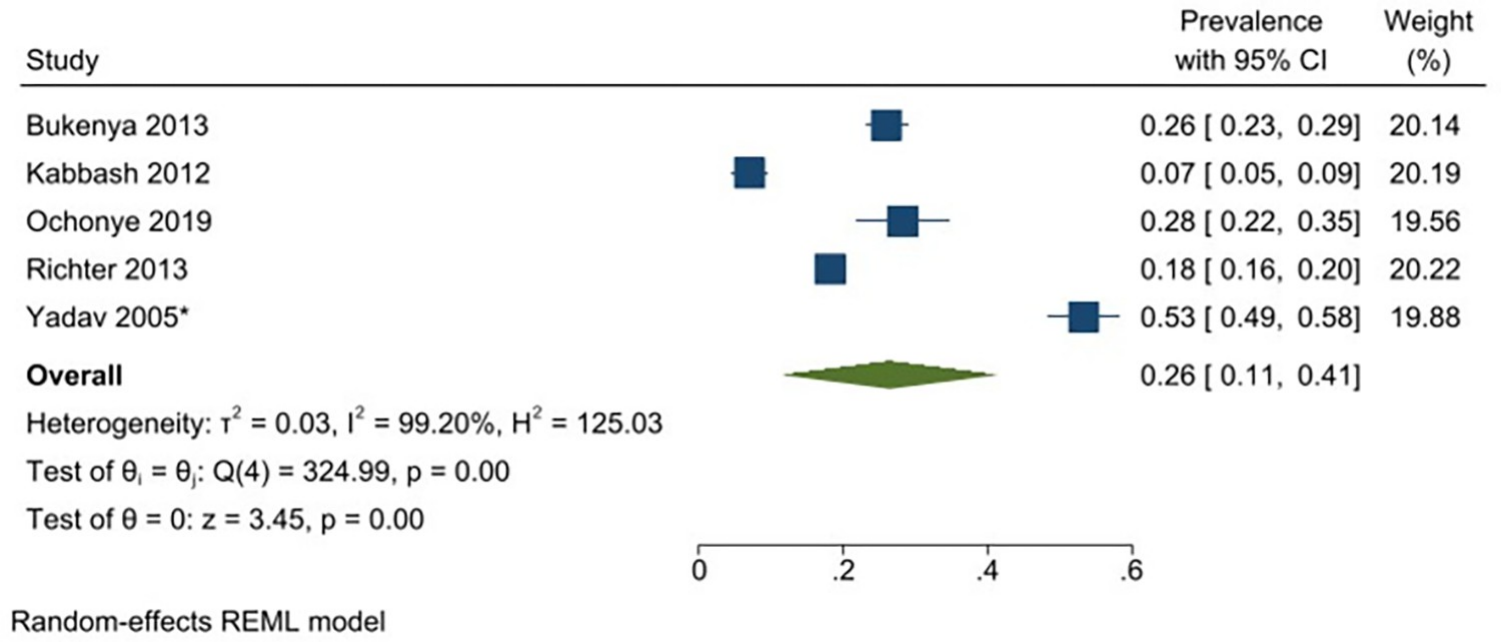
Daily alcohol use—Pooled prevalence estimates for sub Saharan Africa.

**Fig 9:**
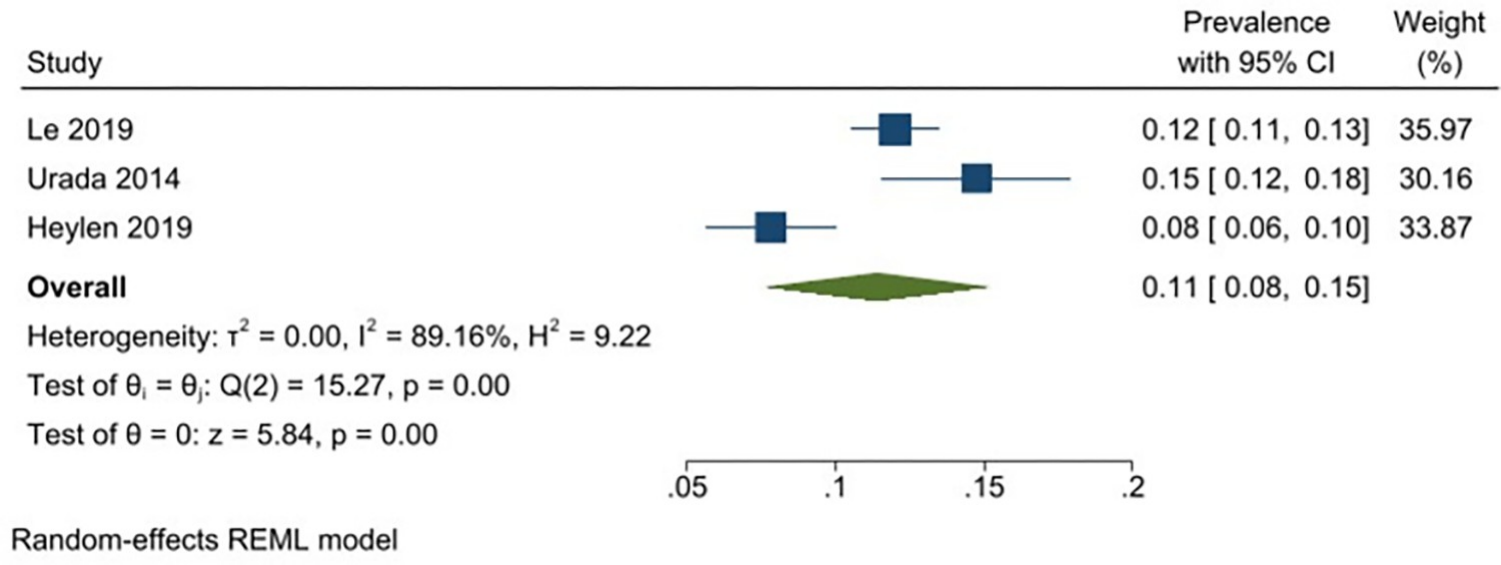
Daily alcohol use—Pooled prevalence estimates for South Asia/ Central Asia/ East Asia and Pacific.

**Fig 10:**
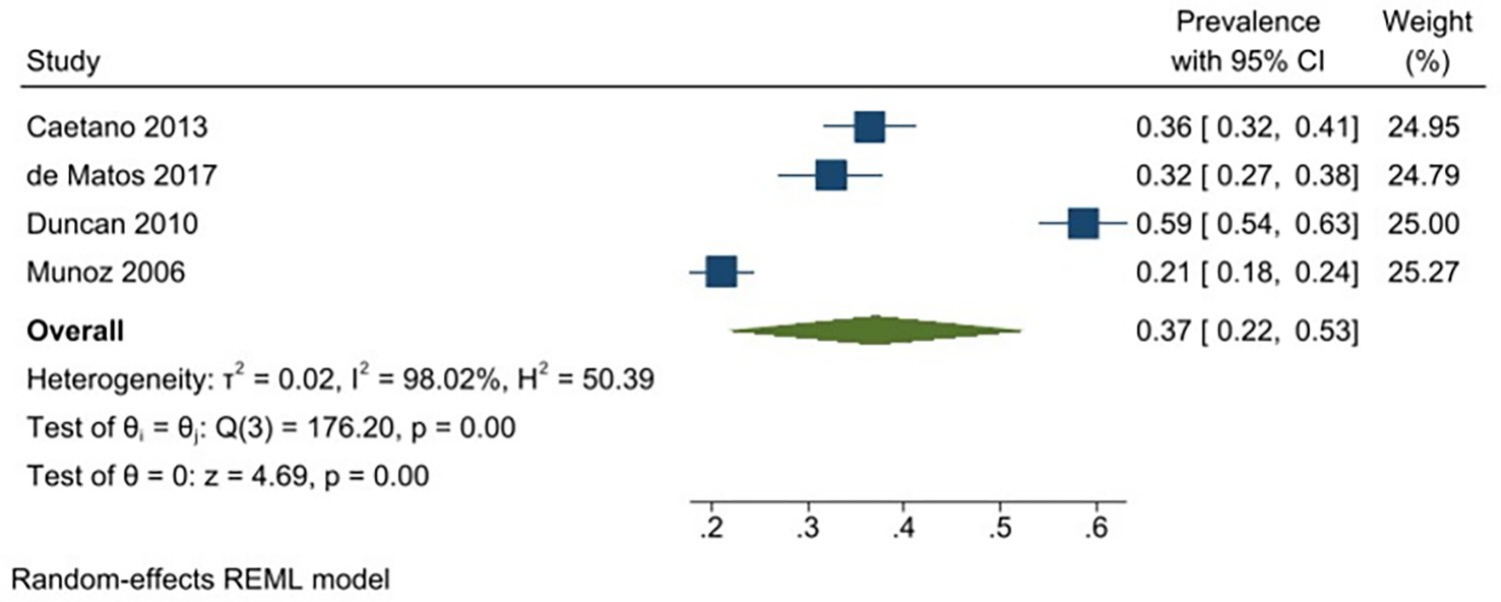
Daily alcohol use—Pooled prevalence estimates for Latin America and the Caribbean.

Twelve studies reported data on daily alcohol use with a reported prevalence that ranged from 8–59% [29, 49, 71, 85, 87, 88, 90, 95, 96, 102, 130, 131]. The pooled prevalence of daily alcohol use among FSWs from LMICs is 26% (95% CI: 17–36%) (*I*^*2*^ = 99.26) (Fig 7). Excluding one study that had selected participants based on HIV-status, the analysis yielded a similar estimated pooled prevalence of 24% (95% CI: 15–33%) (*I*^*2*^ = 99.13). Prevalence estimates were also conducted for each of the different regions, resulting in an estimated pooled prevalence for daily alcohol use of 26% (95% CI: 11–41%) (*I*^*2*^ = 99.20%) in Sub-Saharan Africa (Fig 8); 11.0% (95% CI: 8–15%) (*I*^*2*^ = 89.16%) in South Asia/ Central Asia/ East Asia and Pacific (Fig 9) and 37% (95% CI: 22–53%) (*I*^*2*^ = 98.02%) in Latin America and the Caribbean (Fig 10). All pooled prevalence estimates are summarised in S5 Appendix.

### Associations between harmful alcohol use and other factors

We conducted subgroup meta-analyses to examine associations between harmful alcohol use and factors commonly experienced by FSWs (violence/police arrest, condom use, HIV/STIs, drug use and mental health problems). We only included studies that used a validated alcohol use measurement tool (Tables 2 and 4 –cross sectional studies and Tables 3 and 5 –cohort studies).

**Table 3:** Associations with alcohol use (cohort studies).

Author & Study	Country	Sample	Alcohol use measure	Outcome of interest		Sample size	Events*	Crude Rate ratio (95% CI)	P-value
Chersich (2014)	Kenya	HIV-negative FSWs	Hazardous or harmful drinking (AUDIT score > 8)	Unprotected sex with casual clients		399	90	2.15 (1.06–4.36)	0.03
				Unprotected sex with regular clients		399	156	1.82 (1.20–2.76)	0.005
				Unprotected sex with intimate partner		399	311	1.12 (0.96–1.31)	0.2
White (2016)	Kenya	HIV-positive FSWs	Hazardous or harmful AUDIT score 7–40	Unprotected sex		405	n/a	2.59 (1.7–3.94)	<0.001
Gezie (2015)	Ethiopia	FSWs	Problem drinking–answered yes to one of CAGE questions	Unprotected sex		467	297	1.06 (0.68, 1.68)	0.8
**HIV/STIs**									
Chersich (2014)	Kenya	HIV-negative FSWs	Hazardous or harmful drinking (AUDIT score >8)	HIV incidence	Low risk drinking	399	2	2.82 (0.26–31.09)	0.4
					Hazardous drinking	399	6	10.5 (1.27–87.58)	0.03
					Harmful/dependent drinking	399	1	2.70 (0.17–43.29)	0.5
White (2016)	Kenya	HIV-positive FSWs	Hazardous or harmful AUDIT score 7–40	STI incidence—Diagnosis of vaginal trichomoniasis, gonorrhea, or chlamydia at quarterly exams		405	n/a	2.03 (1.34–3.08)	0.001
Bazzi (2015)	Mexico	FSWs	AUDIT using cut-off >8	STI incidence (chlamydia, gonorrhea, or active syphilis)		185	n/a	2.35 (0.75–7.36)	0.1

*crude rates/no. of events were requested from authors where possible.

**Table 4:** Associations with alcohol use (pooled OR).

Measure	Number of studies included		Pooled unadjusted OR (95% CI)	P-value	studies
Any sexual and/or physical violence		3	2.07 (0.63–6.76)	0.23	Jain 2020
					Semple 2016*
					Chersich 2014*
Inconsistent Condom use		3	1.31 (0.96–1.80)	0.09	Weiss 2016
					Chen 2013
					Semple 2016*
	Excluding studies with HIV negative FSWs only	2	1.48 (0.96–2.27)	0.08	Weiss 2016
					Chen 2013
STI prevalence		4	1.29 (1.15–1.46)	<0.001	Weiss 2016
					Chersich 2014*
					Jain 2020
	Excluding studies with HIV negative FSWs only	2	1.29 (1.14–1.44)	<0.001	Weiss 2016
					Jain 2020
HIV prevalence		3	0.46 (0.12–1.79)	0.3	Nouman 2015
					Weiss 2016
					Couture 2016
Depression		2	1.1 (0.52–2.30)	0.8	Jain 2020
					Coetzee 2018
Illicit Drug use		3	2.44 (1.24–4.80)	0.04	Jain 2020
					Chersich 2014*
					Semple 2016
	Excluding studies with HIV negative FSWs only	2	1.94 (0.90–4.19)	0.1	Jain 2020
					Semple 2016

### Harmful alcohol use, violence and police arrest

In total, ten studies reported associations between violence and alcohol use [27, 28, 42, 50, 63, 84, 92, 96, 113, 118]. Of these, 3 studies used validated tools to measure alcohol use [27, 28, 113] (Table 2) and were included in the meta-analysis with a pooled unadjusted OR of 2.07; 95% CI: 0.63–6.76 (p-value = 0.23) (Table 4). Only one study reported on alcohol use and police arrest [28] (Table 2).

### Harmful alcohol use, condom use and other sexual risk behaviours

Seventeen studies [27, 28, 32, 33, 47, 52, 57, 59, 61, 63, 70, 80, 84, 89, 91, 102, 116] reported associations between condom use and alcohol use. Three studies using a validated alcohol use tool [28, 32, 33] included in a cross-sectional meta-analysis had a pooled unadjusted OR: of 1.31 (95%CI: 0.96–1.80; p-value = 0.09) (Table 4). Three studies [27, 55, 61] (Table 3) were included in a cohort meta-analysis with a pooled unadjusted RR of 1.65 (95%CI 1.01–2.67) for inconsistent condom use and alcohol use (excluding one study that had selected participants based on HIV-status: unadjusted RR: 1.66; 95%CI: 0.69–3.99) (Table 5).

**Table 5:** Associations with alcohol use (pooled RR).

Measure	Number of studies included		Pooled unadjusted RR (95% CI)	P-value	studies
Inconsistent Condom use		3	1.65 (1.01–2.67)	0.04	White 2016
					Gezie 2015
					Chersich 2014*
	Excluding studies with HIV negative FSWs only	2	1.66 (0.69–3.99)	0.3	White 2016
					Gezie 2015
STI incidence		2	2.07 (1.40–3.05)	0.0003	White 2015
					Bazzi 2015

Other associations reported between alcohol use and sexual risk behaviours included unplanned pregnancy [124], reduced use of any contraception [27], increased number of sexual partners [27, 28, 55, 80] and recent anal sex [63, 116]; however there were either insufficient studies to conduct a meta-analysis or validated measures were not used to measure alcohol use.

### Harmful alcohol use, STIs and HIV

Overall 12 studies [27, 32, 33, 60, 62, 63, 75, 78, 97, 101, 108, 113] reported associations between STI prevalence/incidence and alcohol use and one study reported associations with STI symptoms [100]. Three cross sectional studies [27, 33, 113] were included in meta-analysis (pooled unadjusted OR: 1.29; 95%CI 1.15–1.46); similar results were found when one study including only HIV negative FSWs was excluded (pooled unadjusted OR: 1.29; 95%CI: 1.14–1.44) (Table 4). Two cohort studies [55, 62] (Table 3) that reported on alcohol use and STI incidence were included in the meta-analysis, which indicated strong evidence of an association between harmful drinking and STI incidence (pooled unadjusted RR: 2.07; 95% CI: 1.40–3.05) (Table 5).

Nine studies [27, 33, 69, 97, 110, 126, 131] reported on associations between alcohol use and HIV. Meta-analysis among three studies using a validated alcohol use tool found no evidence of an association between harmful drinking and HIV prevalence [33, 73, 97, 126] (Table 4). One cohort study reported an association between hazardous alcohol use and HIV incidence (OR: 10.5; 95%CI: 1.27–87.58) (Table 3) [27].

Two studies reported associations between alcohol use and reduced ART adherence [54, 89], two reported on associations between alcohol use and viral suppression [54, 60] with one reporting an association between harmful alcohol use and viral non-suppression [54]. Due to differences in how these studies measured alcohol and ART adherence/viral suppression, pooled OR were not calculated. One study reported an association between alcohol use and HIV status awareness [53]. There were also associations reported between alcohol use and Hepatitis B [76, 88] and Hepatitis C infection [77, 130]. Two studies reported an association between alcohol use and PrEP use with one finding no association between alcohol use and perceived barriers to oral-PrEP use (based on statements across five domains about why they would not use PrEP) [113] and another finding an association between alcohol use and reduced oral-PrEP adherence [52].

### Harmful alcohol use, depression and illicit drug use

Overall, eight studies [83, 86, 104, 105, 107, 113, 123, 128] reported associations between alcohol use and mental health problems. Two studies [107, 113] that reported on associations with depression were included in a meta-analysis with a pooled unadjusted OR of 1.1 (95%CI: 0.52–2.30). A total of six studies [27, 28, 74, 79, 113, 119] reported on associations between alcohol and any illicit drug use, of which three were included in a meta-analysis with a pooled unadjusted OR of 2.44 (95%CI 1.24–4.80) [27, 28, 113]. When one study including only HIV-negative FSWs was excluded the pooled unadjusted OR was 1.94 (95%CI: 0.90–4.19).

### Alcohol use interventions

Of the four studies reporting on alcohol use interventions, 3 were in sub-Saharan Africa (2 reported on the same study) and 1 was in East Asia and the Pacific. An RCT in Kenya assessed an intervention involving 6 counselling sessions based on the WHO Brief Intervention for Alcohol Use. It reported a statistically significant reduction in alcohol use and binge drinking in the intervention group as well as reductions in violence from clients [39, 40]. An intervention in South Africa [43] assessed an empowerment-based two-session HIV intervention designed to reduce sexual risk, substance use, and violence victimization among at-risk women. At 6 months it found women who received the intervention reported a significantly lower mean numbers of days drinking alcohol in the previous 30 days, were less likely to meet DSM-IV criteria for alcohol dependence, were more likely to report using a condom at last sex with a main partner, and were less likely to report sexual abuse by a main partner in the previous 90 days. An RCT in Mongolia [48] found a reduction in AUDIT score before and after a range of interventions including motivational interviewing, wellness promotion and a relationship-based HIV sexual risk reduction intervention.

## Discussion

In this systematic review and meta-analysis using data from 87 unique studies and including 51,904 FSWs from 32 LMIC countries across all global regions, we found a high prevalence of daily and harmful alcohol use among FSWs associated with a range of risk factors. According to our pooled prevalence estimates two-fifths (41% (95% CI: 31–51%)) of FSWs reported any hazardous/harmful/dependent alcohol use and one quarter (26% (95% CI: 17–36%)) reported daily alcohol use. The global prevalence of alcohol use disorders among women in the general population is 5.1% [132] indicating a significantly higher burden of harmful alcohol use among FSWs. Alcohol use is prevalent during sex work and on entry into sex work, which reflects previous evidence about the availability and normalisation of alcohol in the sex work industry [13, 14]. The high burden of alcohol use has serious health and social implications for FSWs, as excess alcohol use is associated with multiple poor physical and mental health outcomes [133]. Meta-analyses found significant associations between problem alcohol use and inconsistent condom use, increased STI prevalence and incidence and other drug use among FSWs. The associations between harmful alcohol use and wider social and occupational risk factors as well as the high levels of alcohol use during sex work indicate the need to tackle upstream and structural risk factors in future interventions.

Societal standards and norms greatly influence alcohol and drinking patterns. Research from LMICs suggests that policies overseeing the availability of alcohol and legal drinking age are steady predictors of alcohol consumption [134]. Alcohol is widely available in the sex work industry [13] and findings from this review support the fact that alcohol use during sex work is highly prevalent. This correlates with findings from qualitative studies, which report sex workers use alcohol as a way of coping with the challenges of work [14] and due to pressure from clients [135, 136]. In addition, substance use other than alcohol is common during sex work [13, 22, 92], and we found other drug use was associated with harmful alcohol use. Future interventions should consider addressing poly-substance use and tackling social norms around alcohol and other drug use in the sex work environment.

Research from LMICs indicates that alcohol industry marketing has focussed advertising on increasing uptake of drinking among young women [5, 137]. Alongside our findings of a high prevalence of alcohol use on entry into sex work, this indicates that young FSWs should be a target for alcohol interventions to prevent long term alcohol related harms. On a structural level, advocating for wider policy changes around alcohol pricing, accessibility and advertising is also important as these factors have been shown to be crucial in tackling consumption globally [5, 138–140]. In addition, advocating for the de-criminalisation of sex work [12] in the majority of countries globally would make it easier to regulate and improve the safety of the sex work environment.

We did not find associations between alcohol use and violence, however the number of studies included in meta-analysis (n = 3) were limited. Previous associations have been found between alcohol use and intimate partner physical or sexual violence victimization among women in the general population [141]. Interventions addressing violence have been shown to be effective among women in LMICs [142, 143] as well as among FSWs [144], and these should be integrated into future alcohol use interventions. We did not find an association between alcohol use and HIV although this may be due to the limited number of studies reporting alcohol use with a validated tool (n = 3), and in particular lack of longitudinal studies (n = 1), in our meta-analyses. Previous systematic reviews in the general population have reported longitudinal associations between alcohol use and HIV infection [145, 146] and between alcohol use disorders and decreased adherence to antiretroviral therapy and poor HIV treatment outcomes among people living with HIV [147]. Previous quantitative and qualitative research has also indicated that alcohol use may be a barrier to oral-PrEP use [148, 149] including among FSWs [52, 82]. However, the evidence is mixed with other studies reporting no association [113]. Longitudinal studies that measure oral-PrEP adherence, rather than just self-reported oral-PrEP acceptability, are needed to better understand this potential association. We found associations between alcohol use and other sexual risk behaviours including reduced condom use and increased STI infection prevalence and incidence, which echoes findings among women in the general population [150, 151]. Given the well-established links between condom use, STI infection [152] and HIV risk, our findings suggests that alcohol use interventions should be embedded within existing HIV/STI and sexual health services for FSWs, with the view to providing more wholistic integrated services that address women’s physical, psychological and social well-being.

The high levels of alcohol use among FSWs, compared to the general population, and associated risk factors can be considered through a syndemics framework. Syndemic theory aims to identify how the combined effects of health or social epidemics in a population, such as harmful alcohol use and violence, exceeds the sum of their independent components [25, 26] and attempts to identify those most at need as a result of syndemic risks in order to deliver comprehensive and targeted health and social services. To date, the identification of syndemics has largely relied on the use of quantitative methods, which do not go beyond the simple summing of the total number of conditions in individuals [153]. Two previous studies, to our knowledge, among FSWs have examined substance use, violence and HIV from a syndemics perspective, both in relation to oral-PrEP uptake [82, 113]. Only four studies reported on alcohol use interventions for FSWs, all of which are focussed on addressing alcohol use at the individual level only. The syndemic interactions between alcohol use and occupational and socio-economic risk factors associated with sex work are crucial to understand for policy makers developing alcohol use interventions for FSWs, and this should be a key area for future research.

### Limitations

We conducted a comprehensive literature search in line with the PRISMA guidelines, with independent screening and quality appraisal of all studies. Our review captures a broad range of studies from across a variety of geographic regions. Despite this, our review had limitations. Our search was limited to published studies and those written in English and hence we may have excluded important studies. There is a risk of publication bias particularly when interpreting the pooled OR for association, as studies not reporting significant association may be less likely to be published. In addition certain geographic regions are over-represented, such as sub-Saharan Africa, meaning that results may not be generalizable to all settings. We report on unadjusted ORs for the association between harmful alcohol use and potential risk factors, which allows for direct comparisons between studies; however there is a risk that this may have led to over or under-estimated associations. When examining associations, we only included studies in meta-analyses that used a validated alcohol use tool. However, the measures used for associated risk factors such as violence were not all based on validated tools, which could have led to information bias. We included studies in which participants were sampled based on a potential risk factor for alcohol use such as HIV status; however we ran seperate analyses which included and excluded studies that sampled based on HIV status (mainly HIV negative FSWs). We think it is important to show both estimates given the small number of studies eligible for meta-analysis. There was a lack of longitudinal studies (n = 6). Longitudinal studies are necessary to understand the direction of association between alcohol use and common health and social concerns. FSWs are considered a ‘hard to reach population’ due to the stigmatised and illegal status of sex work. As a result FSWs are an inherently difficult population to sample and in many settings, probability sampling is not possible. Due to the sampling strategy of the majority of studies (non-probability sampling) there is a risk that the most vulnerable women were excluded, which may have led to lower estimates of alcohol use. This is a well-established concern in the FSW literature [154] and must be taken into account when assessing study quality, as non-probability sampling techniques such as snow-ball and convenience sampling can lead to selection bias. Techniques such as respondent driven sampling (RDS) that uses a chain referral methodology to collect data from hard-to-reach populations such as FSWs, have been developed to reduce the biases found in standard snowball sampling methods [154, 155]. Future research among FSWs should aim to use sampling methods such as RDS in order to reach the most hidden members of the FSWs population and limit selection bias. Another key limitation was the variability in measurement tools for alcohol use between studies, with many studies not using a validated tool. Even where the same tools were used such as AUDIT, cut-off scores were not always uniformly applied. As a result, comparability and reliability of findings between studies is limited. The use of standardised measurement tools, such as those recommended in the STRIVE ‘Measuring alcohol-related HIV risk’ technical briefing [156], and longitudinal study designs should be prioritised in future research on alcohol use among FSWs.

## Conclusions

This is the first systematic review, to our knowledge, to estimate the prevalence of harmful and daily alcohol use among FSWs in LMICs and to report on associations with common health and social concerns. Our findings suggest that FSWs experience a high burden of daily and problem alcohol use and that harmful drinking is associated with a number of syndemic risk factors including inconsistent condom use, STIs and drug use. There were few alcohol use interventions described; all of these focussed on individual level behaviour change rather than the wider sex work environment that encourages women to engage in excess alcohol consumption. Future research should seek to better understand the syndemic nature of risks, which contribute to high levels of harmful alcohol use among FSWs, and develop wholistic multi-level interventions that address not just individual-level but societal and structural risks such as gender inequality, stigma and poverty.

## Supporting information

S1 Appendix Search strategies. (DOCX)

S2 Appendix Centre for evidence based medicine critical appraisal tool. (PDF)

S3 Appendix Quality assessment of studies. (DOCX)

S4 Appendix List of Stata commands. (DOCX)

S5 Appendix Table of pooled prevalences. (DOCX)

S6 Appendix PRISMA 2020 abstract checklist. (DOCX)

S7 Appendix PRISMA 2020 checklist. (DOCX)
