# Supplementary material for: Alcohol use and associated risk factors among female sex workers in low- and middle-income countries: A systematic review and meta-analysis
Source: PLOS Glob Public Health. 2023 Jun 13;3(6):e0001216. doi: 10.1371/journal.pgph.0001216 (PMC10263362; doi:10.1371/journal.pgph.0001216)
Supplement: S2 Appendix — (PDF) [file pgph.0001216.s002.pdf]

CEBMa
center for
Evidence-Based Management

Critical Appraisal of a Survey

Appraisal questions	Yes	Can't tell	No
1. *Did the study address a clearly focused question / issue?*			
2. *Is the research method (study design) appropriate for answering the research question?*			
3. *Is the method of selection of the subjects (employees, teams, divisions, organizations) clearly described?*			
4. *Could the way the sample was obtained introduce (selection)bias?*			
5. *Was the sample of subjects representative with regard to the population to which the findings will be referred?*			
6. *Was the sample size based on pre-study considerations of statistical power?*			
7. *Was a satisfactory response rate achieved?*			
8. *Are the measurements (questionnaires) likely to be valid and reliable?*			
9. *Was the statistical significance assessed?*			
10. *Are confidence intervals given for the main results?*			
11. *Could there be confounding factors that haven't been accounted for?*			
12. *Can the results be applied to your organization?*			

Adapted from Crombie, *The Pocket Guide to Critical Appraisal*; the critical appraisal approach used by the Oxford Centre for Evidence Medicine, checklists of the Dutch Cochrane Centre, BMJ editor's checklists and the checklists of the EPPI Centre.
