# Supplementary material for: Alcohol use and associated risk factors among female sex workers in low- and middle-income countries: A systematic review and meta-analysis
Source: PLOS Glob Public Health. 2023 Jun 13;3(6):e0001216. doi: 10.1371/journal.pgph.0001216 (PMC10263362; doi:10.1371/journal.pgph.0001216)
Supplement: S3 Appendix — (DOCX) [file pgph.0001216.s003.docx]

## S3 Appendix: Quality appraisal scoring of individual studies included in the review

Table A Quality appraisal scoring

| **Studies** | **1. Did the study address a clearly focused question/issue?** | **2. Is the research method (study design) appropriate for answering the research question?** | **3. Is the method of selection of the subjects (employees, teams, divisions, organizations) clearly described?** | **4. Could the way the sample was obtained introduce (selection)bias?** | **5. Was the sample of subjects representative with regard to the population to which the findings will be referred?** | **6. Was the sample size based on pre-study considerations of statistical power?** | **7. Was a satisfactory response rate achieved?** | **8. Are the measurements (questionnaires) likely to be valid and reliable?** | **9. Was the statistical significance assessed?** | **10. Are confidence intervals given for the main results?** | **11. Could there be confounding factors that haven’t been accounted for?** | **Total score** | **Overall quality assessment** |
| --- | --- | --- | --- | --- | --- | --- | --- | --- | --- | --- | --- | --- | --- |
| Aguayo et al. 2008 | Yes | Yes | Yes | Yes | No | Cannot tell | Cannot tell | No | Yes | Yes | Yes | 5/11 | Moderate |
| Barua et al. 2010 | Yes | Yes | Yes | Yes | No | Cannot tell | Cannot tell | No | Yes | Yes | Yes | 5/11 | Moderate |
| Bautista et al. 2006 | Yes | Yes | Yes | Yes | No | Cannot tell | Cannot tell | No | Yes | Yes | Yes | 5/11 | Moderate |
| Bazzi et al. 2015 | Yes | Yes | Yes | Yes | No | Cannot tell | Cannot tell | Yes | Yes | Yes | Yes | 6/11 | Moderate |
| Bazzi et al. 2019 | Yes | Yes | Yes | Yes | No | Cannot tell | Cannot tell | No | N/A | N/A | Yes | 3/11 | Low |
| Bitty-Anderson et al. 2019 | Yes | Yes | Yes | Yes | No | Yes | Yes | Yes | Yes | Yes | Yes | 9/11 | High |
| Bowen et al.2011 | yes | yes | yes | yes | No | no | Cannot tell | no | no | no | yes | 3/11 | low |
| Bukenya et al. 2019 | Yes | Yes | Yes | Yes | No | Yes | Yes | No | Yes | Yes | Yes | 9/11 | High |
| Bukenya et al. 2013 | yes | yes | yes | yes | No | Cannot tell | Cannot tell | no | yes | yes | Yes | 5/11 | Moderate |
| Caetano et al. 2013 | yes | yes | yes | yes | No | Cannot tell | Cannot tell | No | no | no | yes | 3/11 | Low |
| Carrasco et al. 2019 | Yes | Yes | Yes | Yes | No | Cannot tell | Cannot tell | No | Yes | Yes | Yes | 6/11 | Moderate |
| Chen et al. 2012 | Yes | Yes | Yes | Yes | No | Cannot tell | Cannot tell | No | Yes | Yes | Yes | 6/11 | Moderate |
| Chen et al. 2013 | Yes | Yes | Yes | Yes | No | Cannot tell | Cannot tell | Yes | Yes | Yes | Yes | 7/11 | Moderate |
| Chen et al. 2015 | Yes | Yes | Yes | Yes | No | Cannot tell | Cannot tell | Yes | Yes | Yes | Yes | 7/11 | Moderate |
| Chersich et al. 2007 | Yes | Yes | Yes | Yes | No | Cannot tell | Cannot tell | No | Yes | Yes | Yes | 5/11 | Moderate |
| Chersich et al. 2014 | Yes | Yes | Yes | Yes | No | Yes | Cannot tell | Yes | Yes | Yes | Yes | 7/11 | Moderate |
| Coetzee et al. 2018 | Yes | Yes | Yes | Yes | No | Yes | Yes | Yes | Yes | Yes | Yes | 9/11 | High |
| Conners et al. 2016 | Yes | Yes | Yes | No | No | Cannot tell | Cannot tell | No | Yes | Yes | Yes | 7/11 | Moderate |
| Costa Passos et al. 2004 | Yes | Yes | Yes | Yes | No | Cannot tell | Cannot tell | No | Yes | Yes | Yes | 6/11 | Moderate |
| Couture et al. 2016 | Yes | Yes | Yes | Yes | No | Cannot tell | Cannot tell | Yes | Yes | No | Yes | 6/11 | Moderate |
| Dal Pogetto et al. 2012 | Yes | Yes | Yes | Yes | No | Cannot tell | Cannot tell | Yes | Yes | Yes | Yes | 6/11 | Moderate |
| Damacena et al. 2014 | yes | yes | yes | yes | No | yes | yes | no | yes | no | Yes | 6/11 | Moderate |
| Davis et al. 2017 | Yes | Yes | Yes | Yes | No | Cannot tell | Cannot tell | Yes | Yes | Yes | Yes | 7/11 | Moderate |
| Devine et al. 2010 | Yes | Yes | Yes | Yes | No | Cannot tell | Cannot tell | No | Yes | Yes | Yes | 6/11 | Moderate |
| Duncan et al. 2010 | Yes | Yes | Yes | Yes | No | Cannot tell | Cannot tell | No | Yes | No | Yes | 4/11 | Low |
| de Matos et al. 2017 | Yes | Yes | Yes | Yes | No | Yes | Yes | No | Yes | Yes | Yes | 7/11 | moderate |
| Devoglio et al. 2017 | Yes | Yes | Yes | Yes | No | Yes | Yes | No | Yes | Yes | Yes | 7/11 | Moderate |
| Donastorg et al. 2014 | yes | yes | yes | yes | No | No | Cannot tell | No | yes | yes | yes | 5/11 | Moderate |
| Fang et al. 2007 | yes | yes | yes | yes | No | Cannot tell | Cannot tell | No | yes | no | yes | 4/11 | Low |
| Fawole et al. 2014 | Yes | Yes | Yes | No | No | Yes | Yes | No | Yes | Yes | Yes | 8/11 | High |
| Fearon et al. 2019 | Yes | Yes | Yes | Yes | No | Yes | Yes | No | Yes | Yes | Yes | 7/11 | Moderate |
| Gaines et al. 2013 | yes | yes | yes | yes | No | Cannot tell | Cannot tell | no | no | yes | yes | 4/11 | Low |
| Gezie et al. 2015 | Yes | Yes | Yes | yes | No | Yes | Yes | Yes | Yes | Yes | Yes | 9/11 | High |
| Goldenberg et al. 2012 | yes | yes | yes | yes | No | Cannot tell | Cannot tell | no | yes | yes | yes | 5/11 | moderate |
| Goldenberg et al. 2016 | Yes | Yes | Yes | Yes | No | Cannot tell | Cannot tell | Yes | Yes | Yes | Yes | 6/11 | Moderate |
| Heylen et al. 2019 | yes | yes | yes | yes | No | Cannot tell | Cannot tell | no | yes | yes | yes | 5/11 | Moderate |
| Hong et al. 2007 | yes | yes | yes | yes | No | Cannot tell | Cannot tell | no | yes | yes | yes | 5/11 | Moderate |
| Hooi et al. 2018 | yes | yes | yes | yes | No | Cannnot tell | Cannot tell | no | yes | no | yes | 4/11 | Low |
| Jain et al. 2018 | yes | yes | yes | yes | No | Cannot tell | Cannot tell | no | yes | yes | yes | 5/11 | Moderate |
| Jain et al. 2020 | yes | yes | yes | yes | No | Cannot tell | Cannot tell | yes | yes | yes | yes | 6/11 | Moderate |
| Kabbash et al. 2012 | yes | yes | yes | yes | No | yes | yes | no | no | no | yes | 5/11 | Moderate |
| Karamouzian et al. 2019 | yes | yes | yes | yes | No | Cannot tell | Cannot tell | no | yes | yes | yes | 5/11 | Moderate |
| Kerrigan et al. 2016 | yes | yes | yes | yes | No | yes | yes | no | yes | yes | yes | 7/11 | Moderate |
| Kiene et al. 2019 | Yes | Yes | Yes | Yes | No | Cannot tell | Cannot tell | Yes | Yes | Yes | Yes | 6/11 | Moderate |
| Iaisuklang et al. 2017 | yes | yes | yes | yes | No | Cannot tell | Cannot tell | No | No | No | yes | 3/11 | low |
| Lancaster et al. 2016 | Yes | Yes | Yes | Yes | No | No | No | Yes | Yes | Yes | Yes | 6/11 | Moderate |
| Lancaster et al. 2017 | Yes | Yes | Yes | Yes | No | No | No | Yes | Yes | Yes | Yes | 6/11 | Moderate |
| Le et al. 2019 | yes | yes | yes | yes | No | yes | yes | no | no | yes | yes | 6/11 | Moderate |
| Leddy et al. 2018 | yes | yes | yes | yes | No | Cannot tell | Cannot tell | no | yes | yes | yes | 5/11 | Moderate |
| L´Engle et al. 2014 | Yes | Yes | Yes | Yes | No | Cannot tell | Cannot tell | Yes | Yes | Yes | Yes | 7/11 | Moderate |
| Liao et al. 2012 | yes | yes | yes | yes | No | Cannot tell | Cannot tell | No | No | yes | yes | 4/11 | Low |
| Munoz et al. 2006 | Yes | Yes | Yes | Yes | No | Cannot tell | Cannot tell | No | No | No | Yes | 4/11 | Low |
| Munoz et al. 2010 | Yes | Yes | Yes | Yes | No | Cannot tell | Cannot tell | No | yes | yes | Yes | 6/11 | Moderate |
| Nemoto et al. 2008 | Yes | Yes | Yes | Yes | No | Cannot tell | Cannot tell | No | Yes | Yes | Yes | 6/11 | Moderate |
| Nemoto et al. 2013 | Yes | Yes | Yes | Yes | No | Cannot tell | Cannot tell | No | Yes | No | Yes | 5/11 | Moderate |
| Nouaman et al. 2015 | Yes | Yes | Yes | Yes | No | No | No | Yes | Yes | Yes | Yes | 7/11 | Moderate |
| Ochonye et al. 2019 | yes | yes | yes | no | No | Cannot tell | yes | no | yes | yes | yes | 7/11 | Moderate |
| Odukoya et al. 2013 | yes | yes | yes | yes | No | yes | yes | no | yes | yes | yes | 7/11 | Moderate |
| Pandiyan et al. 2012 | yes | yes | no | yes | No | Cannot tell | Cannot tell | no | no | No | yes | 2/11 | Low |
| Pando et al. 2006 | Yes | Yes | yes | Yes | No | Cannot tell | Cannot tell | Yes | No | Yes | Yes | 6/11 | Moderate |
| Parcesepe et al. 2015 | Yes | Yes | Yes | Yes | No | Cannot tell | Cannot tell | Yes | Yes | Yes | Yes | 7/11 | Moderate |
| Parcesepe et al. 2016 | Yes | Yes | Yes | Yes | No | Cannot tell | Cannot tell | Yes | Yes | Yes | Yes | 7/11 | Moderate |
| Patel et al. 2015 | yes | yes | yes | yes | No | yes | yes | no | yes | yes | yes | 7/11 | Moderate |
| Patterson et al. 2006 | yes | yes | yes | yes | No | Cannot tell | Cannot tell | No | yes | yes | yes | 5/11 | Moderate |
| Persaud et al. 2000 | yes | yes | no | yes | No | Cannot tell | Cannot tell | no | yes | no | yes | 3/11 | low |
| Persaud et al. 1999 | yes | yes | no | yes | No | Cannnot tell | Cannot tell | No | yes | No | yes | 3/11 | low |
| Richter et al. 2013 | Yes | Yes | Yes | Yes | No | Cannot tell | Cannot tell | No | Yes | Yes | Yes | 5/11 | Moderate |
| Sagtani et al. 2013 | Yes | Yes | Yes | Yes | No | Cannot tell | Cannot tell | No | Yes | Yes | Yes | 6/11 | Moderate |
| Salazar et al. 2019 | Yes | Yes | Yes | Yes | No | Cannot tell | Cannot tell | No | Yes | Yes | yes | 5/11 | Moderate |
| Samet et al. 2010 | Yes | Yes | Yes | Yes | No | Cannot tell | Cannot tell | No | Yes | Yes | Yes | 5/11 | Moderate |
| Semple et al. 2015 | yes | yes | yes | yes | No | Cannot tell | Cannot tell | yes | yes | yes | yes | 6/11 | Moderate |
| Semple et al. 2016 | Yes | Yes | Yes | Yes | No | Cannot tell | Cannot tell | Yes | Yes | Yes | Yes | 6/11 | Moderate |
| Semple et al. 2017 | Yes | Yes | Yes | Yes | No | Cannot tell | Cannot tell | Yes | Yes | Yes | Yes | 6/11 | Moderate |
| Servin et al. 2017 | yes | yes | yes | yes | No | Cannot tell | Cannot tell | no | yes | yes | yes | 5/11 | Moderate |
| Singh et al. 2016 | Yes | Yes | Yes | Yes | No | No | No | No | Yes | Yes | Yes | 6/11 | Moderate |
| Strathdee et al. 2008 | Yes | Yes | yes | no | No | Cannot tell | Cannot tell | no | Yes | Yes | yes | 5/11 | Moderate |
| Su et al. 2014 | yes | yes | yes | yes | No | Cannot tell | Cannot tell | yes | yes | no | yes | 5/11 | Moderate |
| Tchankoni et al. 2020 | Yes | Yes | Yes | Yes | No | Cannot tell | Cannot tell | Yes | Yes | Yes | Yes | 7/11 | Moderate |
| Todd et al.  2010 | yes | yes | yes | yes | No | Cannot tell | Cannot tell | No | yes | Yes | yes | 5/11 | Moderate |
| Tran et al. 2014 | yes | yes | yes | yes | No | Cannot tell | Cannot tell | no | yes | yes | yes | 5/11 | Moderate |
| Ulibarri et al. 2014 | Yes | Yes | Yes | Yes | No | Cannot tell | Cannot tell | No | Yes | Yes | Yes | 5/11 | Moderate |
| Urada et al. 2012 | Yes | Yes | Yes | yes | No | Cannot tell | Cannot tell | Yes | yes | yes | yes | 5/11 | Moderate |
| Urada et al. 2014 | Yes | Yes | Yes | Yes | No | Cannot tell | Cannot tell | No | Yes | Yes | Yes | 6/11 | Moderate |
| Verma et al. 2010 | yes | yes | yes | yes | No | yes | yes | no | yes | yes | yes | 7/11 | Moderate |
| Wechsberg et al. 2005 | Yes | Yes | Yes | Yes | No | Cannot tell | Cannot tell | No | Yes | Yes | Yes | 6/11 | Moderate |
| Wechsberg et al. 2006 | yes | yes | yes | yes | No | Cannot tell | Cannot tell | No | yes | yes | yes | 6/11 | Moderate |
| Wechsberg et al. 2011 | Yes | Yes | Yes | Yes | No | Cannot tell | Cannot tell | Yes | Yes | Yes | Yes | 7/11 | Moderate |
| Wechsberg et al. 2008 | Yes | Yes | Yes | Yes | No | Cannot tell | Cannot tell | No | Yes | Yes | Yes | 5/11 | Moderate |
| Wechsberg et al. 2009 | yes | yes | yes | yes | No | Cannot tell | Cannot tell | yes | yes | yes | yes | 6/11 | Moderate |
| Weiss et al. 2016 | Yes | Yes | Yes | Yes | No | Cannot tell | Cannot tell | Yes | Yes | Yes | Yes | 6/11 | Moderate |
| White et al. 2016 | Yes | Yes | Yes | Yes | No | Cannot tell | Cannot tell | Yes | Yes | Yes | Yes | 7/11 | Moderate |
| Wilson et al. 2016 | yes | yes | yes | yes | No | yes | Cannot tell | yes | yes | yes | yes | 7/11 | Moderate |
| Wirtz et al. 2015 | yes | yes | yes | Yes | No | Cannot tell | Cannot tell | No | yes | yes | yes | 5/11 | Moderate |
| Witte et al. 2010 | Yes | Yes | Yes | Yes | No | Cannot tell | Cannot tell | No | No | No | Yes | 3/11 | Low |
| Witte et al. 2011 | Yes | Yes | Yes | Yes | No | Cannot tell | Cannot tell | Yes | Yes | Yes | Yes | 6/11 | Moderate |
| Yadav et al. 2005 | yes | yes | yes | yes | No | Cannot tell | Cannot tell | No | yes | yes | yes | 6/11 | moderate |
| Zhang et al. 2014 | yes | yes | yes | yes | No | Cannot tell | Cannot tell | No | yes | yes | yes | 5/11 | Moderate |
| Zhang et al. 2014 | Yes | Yes | Yes | Yes | No | Cannot tell | Cannot tell | yes | Yes | Yes | yes | 6/11 | Moderate |
| Zhang et al. 2017 | yes | yes | yes | yes | No | Cannot tell | Cannot tel | yes | No | No | Yes | 4/11 | Low |

Table B: Differences in study scores between authors*

| **Study** | **AB** | **OK** | **Question with different score** | **Reason** | **Final score** |
| --- | --- | --- | --- | --- | --- |
| Richter (2013) | 7 | 4 | Q4, 5, 7 | Non-probability sampling methods, no response rate reported | 5 |
| Ulibarri (2014) | 6 | 5 | Q 5 | Non-probability sampling methods reported | 5 |
| De Matos (2017) | 9 | 10 | Q 4, 5 | Non-probability sampling methods | 7 |

***(10% of all study scores were compared)**
