# Supplementary material for: Alcohol use and associated risk factors among female sex workers in low- and middle-income countries: A systematic review and meta-analysis
Source: PLOS Glob Public Health. 2023 Jun 13;3(6):e0001216. doi: 10.1371/journal.pgph.0001216 (PMC10263362; doi:10.1371/journal.pgph.0001216)
Supplement: S4 Appendix — (DOCX) [file pgph.0001216.s004.docx]

# S4 Appendix: Stata commands

*pooled prevalence estimates*

metaprop events samplesize, random

meta set _ES _seES, studylabel(study) studysize(samplesize) eslabel(Prevalence)

meta forestplot, random(reml)

*Pooled OR*

gen lor = log(or)

gen llci = log(lci)

gen luci=log(uci)

meta set lor llci luci, civartolerance(1e-1) studylabel(study) studysize(samplesize)

meta summarize, random(reml) eform

meta forestplot, random(reml) eform

*Pooled RR*

gen lrr = log(rr)

gen llci = log(lci)

gen luci=log(uci)

meta set lrr llci luci, civartolerance(1e-1) studylabel(study) studysize(samplesize)

meta summarize, random(reml) eform

meta forestplot, random(reml) eform
