# Supplementary material for: Alcohol use and associated risk factors among female sex workers in low- and middle-income countries: A systematic review and meta-analysis
Source: PLOS Glob Public Health. 2023 Jun 13;3(6):e0001216. doi: 10.1371/journal.pgph.0001216 (PMC10263362; doi:10.1371/journal.pgph.0001216)
Supplement: S5 Appendix — (DOCX) [file pgph.0001216.s005.docx]

## S5 Appendix: Table of pooled prevalence estimates

|  |  | **Number of studies included** | **Pooled prevalence (95% CI)** | **I^2^** | **Included studies** |
| --- | --- | --- | --- | --- | --- |
| **Any hazardous/harmful/dependent alcohol use problems** |  | 17 | 41% (31-51%) | 98.87% | Tchankoni 2020  Bukenya 2019  Chersich 2014*  Coetzee 2018  Gezie 2015  Kiene 2019  Lancaster 2016  Nouaman 2015  Weiss 2016  White 2016**  Samet 2010**  Chen 2015  Couture 2016  Davis 2017**  Bazzi 2015  Jain 2020  Semple 2016* |
|  | Excluding HIV positive/negative studies | 11 | 43% (31-55%) | 98.96% | Tchankoni 2020  Bukenya 2019  Coetzee 2018  Gezie 2015  Kiene 2019  Nouaman 2015  Weiss 2016  Chen 2015  Couture 2016  Bazzi 2015  Jain 2020 |
| **Harmful/dependent alcohol use only** |  | 5 | 14% (6-22%) | 96.23% | Chersich 2014*  Lancaster 2016**  White 2016**  Chen 2015  Samet 2010** |
| **Alcohol use Sub-saharan Africa** |  | 10 | 38% (27-48%) | 98.11% | Bukenya 2019  Chersich 2014*  Gezie 2015  Kiene 2019  Lancaster 2016**  Coetzee 2018  Nouaman 2015  Tchankoni 2020  Weiss 2016  White 2016** |
|  | Excluding HIV positive/negative only studies | 7 | 39% (26-52%) | 98.69% | Bukenya 2019  Gezie 2015  Kiene 2019  Coetzee 2018  Nouaman 2015  Tchankoni 2020  Weiss 2016 |
| **Alcohol use** **S****outh Asia/ Central Asia/ East Asia and Pacific** |  | 4 | 47% (17-77%) | 99.25% | Samet 2010**  Davis 2017**  Couture 2016  Chen 2015 |
|  | Excluding HIV positive/negative studies | 2 | 68% (36-101%) | 98.62% | Couture 2016  Chen 2015 |
| **Latin America and the Caribbean** |  | 3 | 44% (18-69%) | 98.94% | Bazzi 2015  Jain 2020  Semple 2016* |
|  | Excluding HIV positive/negative studies | 2 | 33% (7-59%) | 97.70% | Bazzi 2015  Jain 2020 |
| **Daily alcohol use** |  | 12 | 26% (17-36%) | 99.26% | Bukenya 2013  Kabbash 2012  Ochonye 2019  Richter 2013  Le 2019  Urada 2014  Heylen 2019  Caetano 2013  de Matos 2017  Duncan 2010  Yadav 2005*  Munoz 2006 |
|  | Excluding HIV positive/negative only studies | 11 | 24% (15-33%) | 99.13% | Bukenya 2013  Kabbash 2012  Ochonye 2019  Richter 2013  Le 2019  Urada 2014  Heylen 2019  Caetano 2013  de Matos 2017  Duncan 2010  Munoz 2006 |
| **Daily alcohol use sub-Saharan Africa** |  | 5 | 26% (11-41%) | 99.20% | Bukenya 2013  Kabbash 2012  Ochonye 2019  Richter 2013  Yadav 2005* |
|  | Excluding HIV positive/negative only studies | 4 | 20% (10-29%) | 97.79% | Bukenya 2013  Kabbash 2012  Ochonye 2019  Richter 2013 |
| **Daily alcohol use** **South Asia/ Central Asia/ East Asia and Pacific** |  | 3 | 11% (8-15%) | 89.16% | Le 2019  Urada 2014  Heylen 2019 |
| **Daily alcohol use** **Latin America and the Caribbean** |  | 4 | 37% (22-53%) | 98.02% | Caetano 2013  de Matos 2017  Duncan 2010  Munoz 2010 |
| *HIV negative FSWs only  **HIV positive FSWs only | | | | | |
