# Supplementary material for: Alcohol use and associated risk factors among female sex workers in low- and middle-income countries: A systematic review and meta-analysis
Source: PLOS Glob Public Health. 2023 Jun 13;3(6):e0001216. doi: 10.1371/journal.pgph.0001216 (PMC10263362; doi:10.1371/journal.pgph.0001216)
Supplement: Attachment — Submitted filename: Response to reviewers.docx [file pgph.0001216.s008.docx]

Department of Global Health and Development

London School of Hygiene and Tropical Medicine

15-17 Tavistock Place

London WC1H 9SN

4^th^ October 2022

Dear Editors,

We thank the reviewers for their time and for their helpful feedback on the manuscript. We have responded to each of the comments and suggestions and made relevant amendments to the manuscript. We hope these changes have improved the final manuscript. A point by point response to reviewers are included below.

We look forward to hearing from you regarding this manuscript.

Yours sincerely,

Alicja Beksinska

On behalf of co-authors

Journal requirements

1. Please provide separate figure files in .tif or .eps format and ensure that all files are under our size limit of 10MB.

For more information about how to convert your figure files please see our guidelines: <https://journals.plos.org/globalpublichealth/s/figures>

Response: Please see separate files uploaded.

2. Please include your main tables as part of your main manuscript and remove the individual files.

Response: Tables included in main text.

3. Please ensure that you refer to Figure 10 in your text as, if accepted, production will need this reference to link the reader to the figure.

Response: The figures had been incorrectly referenced in the text– these are now all labelled correctly and referenced in the text.

4. We noticed that you used “unpublished” in the manuscript. We do not allow these references, as the PLOS data access policy requires that all data be either published with the manuscript or made available in a publicly accessible database. Please amend the supplementary material to include the referenced data or remove the references.

Response: We have amended this as this study has since been published.

5. We have noticed that you have uploaded Supporting Information files, but you have not included a list of legends. Please add a full list of legends for your Supporting Information files after the references list.

Response: This has now been amended and a list of legends has been included at the end of the references list.

Please use the space provided to explain your answers to the questions above. You may also include additional comments for the author, including concerns about dual publication, research ethics, or publication ethics. (Please upload your review as an attachment if it exceeds 20,000 characters)

Reviewer #1: Manuscript review: PGPH-D-22-01100

Overall assessment
This is a promising and important study about a relevant and current topic in global public health. Overall, the study is methodologically sound, adheres to the current standards of a modern epidemiological study, and merits publication, but some sections, especially methodology and results, are too brief, and need to be expanded and more detailed. The language of the study appears to reflect that the main writer(s) are not native speakers of English and are new to scientific writing. There are minor misconceptions regarding study designs and the measures of disease frequency and effect they produce. The quality of the writing can be improved, as the manuscript in its present form does not reflect the quality and amount of work that was put in this research project. The senior author, especially if he/she is a primary speaker of English, should review and edit carefully. The junior authors of the study seem to need guidance and the manuscript will benefit greatly from feedback from a more senior researcher, which can pick up on several areas for improvement which this reviewer noticed, but which are not the task of a reviewer to correct.

Response: We thank the reviewer for their feedback. All the study authors are primary speakers of English and have extensive experience in writing scientific papers. If there are particular areas where the reviewer feels that understanding of the content of the manuscript is impaired by the language used then we would be happy to review this.

This study would also have benefitted from a narrower aim. The decision to include in the same systematic review the aim to estimate prevalence of problem alcohol use among FSWs and the association of alcohol use disorder with other exposures experienced by FSWs (HIV, STIs, violence, etc.), made this systematic review and the manuscript unnecessarily more complex, especially because the kind of study designs that answers those questions are distinct. Cross-sectional design is the ideal design to estimate prevalence, while case-control studies do not allow to calculate prevalence, since we don’t have access to the source population that produced the cases. This reviewer questions the decision to include experimental studies, despite the authors attempt to justify that they were including experimental studies with baseline assessments. An experimental study is an artificial situation that is designed to answer a particular question, and often involves populations and contexts that make those studies feasible. This reviewer would argue that any experimental study included provides a biased estimate of prevalence of alcohol use among FSWs, given the inherent nature of experimental studies, which have strict inclusion criteria and willingness of participants to receive intervention being proposed by the investigators. Given the potential of selection bias inherent to any experimental studies, this reviewer would argue against the inclusion of any experimental design in this present study. In retrospect, the authors probably should have concentrated in providing an estimate for the prevalence of the distinct forms of alcohol use, while studying the association of alcohol use with other exposures/outcomes of interest to a separate study. The systematic review of the association of alcohol use with other risk factors lived by FSW should have been saved for a separate study.

Response: We thank the reviewer for this feedback. However, we disagree that the aim of the study is too broad and makes the study too complex. It is common practice for systematic reviews to examine both the prevalence and associated risk factors of a given health problem. This includes systematic reviews among FSW populations published in PLOS journals (e.g. Beattie et al(1) published in PLOS Medicine). The majority of studies among FSWs do not use validated tools to measure alcohol use, meaning there were only a limited number of studies available to measure associations with alcohol use. We do not think this warrants a separate review; instead it was necessary and relevant to include associations with other risk factors within this paper. Finally, our review was informed by syndemic theory, which emphasises the interconnected and multiplicative nature of health and social issues. This is particularly important in a key population such as FSWs, who experience an intersection of health and social inequalities (lines 148-153, introduction). Based on a syndemics framework we felt it was important to consider not just the burden of alcohol use in a silo, but the key associated risk factors.

The reviewer has commented on the inclusion of case-control studies within a systematic review. Although there were no case control studies included in the review, we think it is important they were eligible for inclusion as they could have been a source for data on associations. Although there are differences of opinion in the literature on systematic reviews as to whether different study designs should be included within the same review, we would argue that it is an accepted method in systematic reviews to include all types of observational studies(2) when examining the burden of a particular health condition. We refer to Beattie et al. systematic review in which all observational studies (including case-control studies) were eligible for inclusion(1). Another study, reporting on the burden of HIV among FSWs published in the Lancet Infectious Diseases states ‘studies of any design were included that measured the prevalence or incidence of HIV(3)’. Examples such as these two systematic reviews, published in high impact journals, were used as a guide for our methodology. Given the paucity of studies among FSWs measuring alcohol use with a validated tool we felt it was important to consider all observational studies.

The reviewers have stated that “any experimental study included provides a biased estimate of prevalence of alcohol use among FSWs, given the inherent nature of experimental studies, which have strict inclusion criteria and willingness of participants to receive intervention being proposed by the investigators”. We would argue that using baseline estimates from experimental studies is an accepted practice in systematic reviews. We refer to previous systematic reviews among FSWs that include baseline measures from experimental studies (3, 4) and include all quantitative study designs. In response to the point on bias, we acknowledge that most studies among FSWs are at high risk of selection bias because FSWs are a hard to reach and hidden population. As a result FSWs who take part in studies are likely to differ from those who do not take part, and this is well documented in the FSW literature (5). We agree that studies with FSWs will have inherent biases and have expanded on this in the limitations section of our discussion (lines 206-219, discussion). Given the limited number of studies among FSWs reporting on alcohol use with validated measures it is important we did not overly limit our inclusion criteria.

In trying to include everything in the same study, several methodological aspects of study design and results did not get the space they deserved. One area of particular concern, and which should have received great deal of attention, is a comprehensive and thoughtful discussion about the potential for bias, especially selection bias, in each original studies included in the meta-analysis. Despite the authors argument that they used a tool to critically appraise the original studies, the report that 27 studies scored as high quality, 62 scored as moderate and 9 scored as weak, is surprising. Just the fact that most of the original studies did not use probabilistic sampling already makes them at very high risk of selection bias, and that on itself would be enough to make the evidence provided by a study weak, at best.

Response: We thank the reviewer for their feedback on bias. We have amended the manuscript to include a more detailed discussion about the risk of selection bias (line 206-219, discussion). We acknowledge that the majority of studies included used non-probability sampling and are therefore at higher risk of bias. FSWs are an inherently difficult population to sample. It is important to acknowledge that a major challenge of sampling hidden populations such as FSWs is due to the stigmatised and illegal status of sex work(5) which make them difficult to access and often unwilling to participate in research. As a result there are few studies among FSWs that have used probabilistic sampling methods. This inevitably introduces a risk of bias as the most vulnerable FSWs may be more likely to be excluded from research. Although it is essential this is acknowledged in the study limitations, we would also argue that many of the sampling techniques which were used are the best available for a hidden population. This is widely discussed in the FSW literature(5-9). Some of the studies use either respondent driven sampling (RDS) or variations on RDS such as snowball sampling. RDS is considered in the literature to be an efficient and robust means to sample hard-to-reach populations at risk for HIV(6). RDS incorporates numerous theoretical assumptions borrowed from several disciplines, including network theory, physics, statistics and mathematics, to reduce the biases found in standard sampling methods. We have expanded on this discussion in our limitations section, and included recommendations for future studies among FSWs to prioritise the use of RDS as a sampling method.

The critical appraisal tool is a basic tool to screen studies for inclusion in the systematic review, but should not serve as the only measure of the potential for bias in the estimates used in the meta-analysis. Besides, item 5 of the appraisal tool “Was the sample of subjects representative with regard to the population to which the findings will be referred?” was answered “yes” for almost all the studies. That is not correct, when item 4 “Could the way the sample was obtained introduce (selection)bias?” was also answered “yes” for nearly all the studies. If item 4 is a “yes”, then item 5 is automatically a “no”, or “unknow”, at best. If there is potential for selection bias, we can’t know if the study sample is representative of the target population; and with the use of non-probability sampling, that is almost certainly a “no” on item 5 for nearly all the studies.

Response: We thanks the reviewer for their comments on the use of the critical appraisal tool. As a result of this feedback we will amend our CEBM item 5 responses to ‘no’ as we do understand the concerns raised by the reviewer with regard to bias (see appendix 2 and 3). Having amended this the majority of studies still score as moderate quality (n=78) (lines 295-296, results).

Nearly all the original studies included in this meta-analysis are at great risk of bias and the authors do not do enough to address that fundamental issue with this meta-analysis. This fundamental risk of bias in the pooled estimate, originating from the individual studies, needs to be addressed in the introduction, methods, results, and discussion. The authors touch on that, but it requires a more comprehensive treatment. This writer points the authors to the PRISMA statements items (below) that require a more comprehensive approach to the risk of bias in the original studies.

Response: We have addressed concerns about selection bias in the limitations section of the discussion (lines 206-219, discussion). To avoid repetition, we do not think this needs to be repeated in the introduction, method and results. We would again refer to previously published literature where risk of bias is addressed primarily in the limitations section of the discussion(1, 3).

Lastly, any meta-analysis needs to address the risk of publication bias, especially in a topic of studies in LMICs. The authors did not address sufficiently the risk of publication bias and that should be included in the methods, results, and discussion section, including producing and reporting a funnel plot. Similarly, this writer points the authors to the PRISMA statement items that require a more comprehensive treatment of the risk of publication bias in a systematic review and meta-analysis.

Response: We thank the reviewer for this comment. We have included a comment in the limitations section of the study on the risk of publication bias (lines 173-175, discussion).

Specific comments
TITLE
“low-and middle income countries”
Correct way to write is “low- and middle-income countries”.

Response: This has been amended (lines 2-5, title page)

INTRODUCTION

“In recent years alcohol use has been increasing in many LMICs.”
Needs reference.

Response: This has been referenced.

“which corresponds to the AUDIT tool and some other alcohol use tools.”
Not professional English; “some other” is not a professional and scientific statement in this case.

Response: We have removed ‘some’.

METHODS
Methods section needs to be expanded and more detailed. In particular the following items on the PRISMA statement:
• 11 Specify the methods used to assess risk of bias in the included studies, including details of the tool(s) used, how many reviewers assessed each study and whether they worked independently, and if applicable, details of automation tools used in the process.,
• 14 Describe any methods used to assess risk of bias due to missing results in a synthesis (arising from reporting biases), and
• 15 Describe any methods used to assess certainty (or confidence) in the body of evidence for an outcome.

Response

- 11 We have used the CEBMa tool to assess risk of bias, as this includes questions on bias. This is detailed in our methods and in appendix 2 and 3. In addition we have made amendments to our responses to the question on selection bias (item 5 CEBMa) based on feedback from the reviewer. In our methods section under quality assessment we have detailed how many reviewers assessed each study, what percentage of studies were assessed by both reviewers and how discrepancies in reviewers assessments were addressed. We have additionally included a table in Appendix 3 which indicates discrepancies in scoring between reviewers with final agreed scores. We have also detailed how we excluded from the meta-analysis, studies that selected participants based on harmful drug or alcohol use .We also provide separate estimates for studies that selected participants based on HIV status.
- 14 We have included a sentence on publication bias in the limitations section of the discussion (lines 189-191, discussion).
- 15 We have reported confidence intervals for pooled prevalence estimates and OR/RR associations.

“This review included studies which reported any measure of prevalence or incidence of alcohol use on the basis of a clinical interview, self-reported or clinical examinations among FSWs even if sex workers were not the main focus of the study.”

“The following study designs were included: cross-sectional survey, case–control
study, cohort study, case series analysis, or experimental study with baseline
measures for alcohol use.”

Case-control studies, case series, and clinical trials do not allow for calculation of prevalence, nor incidence (theoretically you can estimate incidence in a clinical trial, but not with the outcome of interest in this study). The authors need to clarify the inclusion criteria or explain the contradiction that they intended to include only studies with measure of prevalence or incidence of alcohol use disorder and yet list case-control, case series, or an experimental study design. While including case-control studies or cohort studies would have helped estimate strength of association of alcohol use with violence, STIs, inconsistent condom use, etc, those studies would not meet the including criteria of reporting prevalence or incidence of alcohol

Response: Our study aims were to examine the prevalence of alcohol use *and* associated risk factors. We have amended the inclusion criteria to include ‘associations with alcohol use’ to ensure this is stated more clearly (line 193, methods).

“Centre for Evidence-Based Management (CEBM) Critical Appraisal for Cross-Sectional Surveys Tool”
They should name and abbreviate correctly as Center (notice the American spelling on the name on supplement 2), and abbreviate as CEBMa, otherwise it is confused with the Centre for Evidence-Based Medicine (CEBM) at Oxford (<https://www.cebm.ox.ac.uk/resources/ebm-tools/critical-appraisal-tools>).

Review: Thank you for this comment. We have amended this as suggested.

“Through narrative synthesis of available measures and known validated tools we selected a range of measures of alcohol use and harmful alcohol use for meta-analysis.”
Can they clarify? What do they mean?

Response: As is detailed in table 1 we extracted information on any measure of alcohol use. We summarised the alcohol measures reported in our narrative synthesis e.g. X number of studies used AUDIT and X number of studies reported on daily alcohol use. Given that validated tools are more reliable estimates of alcohol use, we first selected studies reporting on alcohol use using a validated tool for meta-analysis. The only other estimate of alcohol use that was consistently reported across studies was ‘daily alcohol use’ and therefore we selected this outcome for meta-analysis. We were able to determine this through our narrative synthesis. We have amended this sentence in the methods for clarity (lines 246-247, methods).

They have to describe how they extracted information on association of alcohol use with other outcomes. This needs to be greatly expanded in the methods section and the discussion along the lines of the PRISMA items pointed out above.

Response: All included studies were reviewed and information on associations with alcohol use was extracted by reviewers and reported in narrative synthesis. All included studies using validated tools had data on associations with alcohol use extracted into a formal extraction table (Table 3 and 4). We have added a few sentences into the methods to clarify this (line 258-260, methods).

The authors should mention which Stata 16 command they use to perform meta-analysis of proportion and odds-ratio.

Response: We have included our Stata 16 commands as an appendix (please see appendix 4)

RESULTS
The results need to be improved concerning the following PRISMA statement items: 18 (Present assessments of risk of bias for each included study.), 21 (Present assessments of risk of bias for each included study.), and 22 (Present assessments of certainty (or confidence) in the body of evidence for each outcome assessed.)

“The initial electronic search yielded 416 results, with 19 additional studies identified through reference list screening and online searches.”
The methods section did not say anything about screening reference list and on-line search. That is not a “systematic review” approach, and those methods should not have been used, since another investigator would not be able to reproduce the same search. “Augmenting” the systematic review by combing through references or the internet defeats the purpose of a systematic review, which is to produce a comprehensive, reproducible literature search.

Response: We thank the reviewer for this query. The authors would like to refer the reviewer to the latest PRISMA 2020 flow diagram which includes “Identification of studies via other methods” and specifically includes ”citation searching” as a valid method for identifying relevant studies(10). This is standard practice and considered important for both increasing the number of relevant studies for meta-analysis and reducing the risk of publication bias whereby only studies that appear in database searches are considered eligible. We have amended the methods to ensure this is clear in the text (lines 188-189, methods) as this had already been detailed in the PRIMSA flow chart. In addition we refer to the Cochrane guidelines chapter 6 on ‘handsearching’ and ‘reference list searching’ as a useful adjunct to electronic database searches. (<https://handbook-5-1.cochrane.org/chapter_6/6_2_2_1_handsearching.htm> and <https://handbook-5-1.cochrane.org/chapter_6/6_2_2_5_other_reviews_guidelines_and_reference_lists_as.htm> )

Table 1, column 1: did they mean author and year?

Response: This has been amended.

Table 1, column 4 “Sampling procedure”, the term non-random sampling is not descriptive; did they mean non-probabilistic or non-probability sampling? That is a more appropriate term than “non-random sampling”. What do they mean by non-random? All the other methods of sampling listed (venue-based, convenience, snowball, respondent-driven, purposive, targeted, etc.) are non-probabilistic/non-probability (“non-random”) sampling techniques. Please clarify what they mean by “non-random sampling” and why they chose to use that term for some studies and not for others, since nearly all the studies used non-probability sampling. I am guessing the authors of the original article did not describe the sampling procedure used.

Response: “Non-random sampling” refers to “non-probabilistic sampling”. Some studies did not describe the sampling procedure and others used the term non-random. We have amended the description to “non-probabilistic sampling” to improve the uniformity of the descriptions as per the reviewers recommendations (lines 280-285, results).

Also, there is a heterogeneous use of terms. All the sampling techniques described, snowball, respondent-driven, target, purposive, venue-based, etc, despite their fancy names, amount to non-probability sampling, also referred to as convenience sampling.

The authors should consider categorizing the type of sampling used in each study as either probability sampling, and then describe the type of probability sampling technique used (simple random, stratified, cluster, multistage, systematic, complex), or non-probability sampling, and then list the type used.

Response: These are direct terms used by the study authors to describe their sampling methods. We think it is important to report these in the table. We have categorised the type of sampling more clearly in our results (lines 280-285, results).

“A variety of measures on drinking prevalence and frequency were reported with the
majority of studies not using a validated tool to assess alcohol use.”
This sentence is too vague. If this is being presented in results, stating “the majority” is not sufficient; how many studies and what percentage of studies did not use a validated tool? For example, of the N studies included in this meta-analysis, n (%) used a validated tool to assess alcohol consumption, while n (%) did not.

Response: We have amended this sentence to report on the percentage of studies not using a validated tool (line 6, results). This was also detailed in the paragraph below (title: Prevalence of alcohol use reported using validated tools, line 22-36, results) where we report the number of studies that use a validated tool. We conducted a meta-analysis of studies using a validated tool (AUDIT, CAGE, CIDI) and separately of studies reporting daily alcohol use as this was the only consistently reported measure in terms of time-frame and is a useful indicator for policy makers about the frequency of alcohol use in this population. Details of included studies are reported in the paragraphs on ‘daily alcohol use’ and ‘harmful alcohol use’ in the results.

Table 1, Column 6, Method of assessing outcome
Fawole (2004) “questionnaire”, this is not sufficiently descriptive.
Leddy (2018) “survey”
Ochonye (2019) “Semi-structured interviewer administered
Response: This is what was reported in the individual studies – no further description is available.

Pattern of alcohol use
This subsection of the results section reporting of the patterns of alcohol seems redundant. The authors set out to study hazardous, harmful, and alcohol dependence and should have concentrated on the defined outcomes for this study. Yet they provide a description of using alcohol while engaging in sex work.

Response: We thank the reviewer for this feedback. However, we disagree that this is redundant. Narrative synthesis is important to describe the range of alcohol use measures reported and the heterogeneity in measures. As highlighted in the discussion, this finding indicates the need for future research among FSWs to use validated measures as the current heterogeneity in alcohol use measures makes it difficult to compare across studies. Given the high frequency of alcohol use during sex work and the impact on associated risks such as condom use and violence, we think it is important information for policy makers and researchers working with sex workers to report on the prevalence of alcohol use during sex work.

Table 2 seems redundant, since result of meta-analysis is already presented as a forest plot, and increases the size of publication. The authors should consider if the information in table 2 is not already present in the forest plots and other parts of the results.

Response: Table 2 has been included as an appendix (appendix 5) for readers who may find the information useful in table format.

Harmful alcohol use
“A meta-analysis was conducted with seventeen unique studies that used a validated tool (excluding studies which included only substance using FSWs) and estimated the pooled prevalence of any harmful alcohol use to be 41% (31-51%).”

If the numbers in parenthesis are the confidence interval, the author should have written (95% CI, 31% to 51%), or indicated that it is the range of values; just writing the limits of the confidence interval in the narrative portion is not the standard for reporting, otherwise the reader does not know what is being reported between parenthesis.

The same comment goes for all the numbers in parenthesis in the section “Harmful alcohol use”.

Response: Thank you, we have amended this.

One important portion of the results section is that the authors need to provide a funnel plot to show that there was no publication bias. This can be accomplished with meta funnelplot command in Stata.

Response: Thank you for this suggestion however we do not think that a funnelplot is necessary for our systematic review. Our understanding of publication bias is that it is more likely to occur when the outcome of a research study biases the decision to publish it – this is of greater concern in intervention studies than in observational studies. Although publication bias is discussed in our limitations, given that this is a systematic review reporting primarily on pooled prevalence from observational studies, there is less risk of publication bias as there is limited incentive for authors to not publish data on prevalence of alcohol use. We do report on associations data which is at greater risk of publication bias; however the number of studies included in meta-analysis is insufficient and therefore the power is too low for a funnel plot to distinguish chance from real asymmetry. (https://handbook-5-1.cochrane.org/chapter_10/10_4_3_1_recommendations_on_testing_for_funnel_plot_asymmetry.htm )

Alcohol use interventions
Unclear why there is a section on alcohol use interventions. At any point during the introduction and methods the authors indicate that this study was interested in reporting on alcohol use interventions.

Response: Although this was not the primary focus of the review we thought it was important for researchers and policy makers to be aware of the number of intervention studies addressing alcohol use for FSWs. As we used baseline measures from experimental studies, these studies were eligible for inclusion, which is noted in our methods. Given that the number of intervention studies is so small, this would not warrant a separate review. Thus, we think it is important they are reported in our systematic review to highlight the need for alcohol use interventions for FSW populations.

DISCUSSION
The main focus of this study, prevalence of alcohol use among female sex workers, is not a simple outcome to ascertain. The same can be said about the other outcomes (STIs, HIV/AIDS, violence, etc.). Estimates obtained in the original studies are at high risk for selection and information bias, and confounding. Although the investigators did employ a tool to rate the quality of the study, special attention and space in the article should be dedicated to discussing the risk of bias in the original estimates.

Although the authors do mention the fact that most studies used non-probability sampling methods in the discussion section, they need to greatly expand on that portion of discussion since this is the foundation of the validity of all the work put in this study.

They need to discuss the non-probabilistic sampling procedures used in this “hard-to-reach” population and implications to bias and accuracy of estimates of prevalence.

The authors also need to provide an assessment about the risk of bias and confounding for the association of alcohol use with other exposures/outcomes in the original studies.

A discussion about publication bias needs to also be included.

Response: We thank the author for this feedback. We have expanded on our limitations paragraph in the discussion and hope this addresses the above issues (line 185-219, discussion).

STYLE
Manuscript should have been submitted double-spaced.
Reference should be in brackets; lines should be numbered.
Formatting of paragraphs need to be fixed. Too many paragraphs are making it difficult to read.
The authors don’t need to reference each study included in the meta-analysis when reporting the results in the body of the text; some statements in the result section are followed by a long list of references. That is not necessary. If the information is relevant it should be in the table describing the studies and the narrative portion of results should make reference to the table.
Not sure why the editorial staff accepted this manuscript that does not follow the basic rules of submission to PLOS GPH.

Response: We have addressed these formatting issues and reduced the number of paragraphs in the introduction. However, the rest of the manuscript has been reviewed and we think that the current number of paragraphs are necessary to ensure paragraphs are not excessively long, particularly given the volume of additional content being added in the review process. With regards to references, we have followed examples based on previously published systematic reviews on PLOS journals which do include references in the body of the text. Could the editors clarify what is the preferred format?

## REVIEWER 2

Reviewer #2: The authors conducted a systematic review and meta-analysis of alcohol use among female sex workers in LMICs. Their work is well-written and results are presented clearly.

I have a few comments that should be addressed before publication of this work.

(1) The authors used the Appraisal tool for Cross-Sectional Studies to classify study quality. Please list a few quality indicators to give the reader an idea on what criteria are important in this quality characterization. The classification thresholds (0–4,5–7,8–11) were chosen by the authors not by CEBM, right? This should be clarified too.

Response: We thank the reviewer for their feedback on this. We have included the full questions for the CEBMa quality tool in appendix 2. We have summarised a few key criteria in the methods in paragraph 2 on ‘Quality Assessment’ (line 225-227, methods). We used cut off criteria based on a previous systematic review on mental health problems among FSWs published in PLOS medicine which had also used the CEBMa tool (4). Given that this systematic review was in a FSW population, and had used the same tool, we chose to use the same classification thresholds.

(2) In the study quality scoring “authors compared 10% of the results of scoring and discussed disagreements in scoring to ensure uniformity in the quality assessment process.” Can the authors please comment on score differences? Some of the used criteria may not be completely objective and it would be of interest to report score differences.

Response: We have included a table in Appendix 3 (quality assessment) which details score differences. The main differences observed was for questions 4 and 5 (was there selection bias and was the sample representative?). After further discussion between authors and based on reviewer feedback, as the majority of studies used non-probability sampling methods there was likely to be risk of selection bias and risk of the sample not being representative for almost all studies. The issue of selection bias due to sampling difficulties in a ‘hidden’ and ‘hard to reach’ population such as FSWs has since been expanded upon in the limitations section of the discussion following reviewer 1 comments (lines 206-219, discussion).

(3) Did the authors use “study quality” to weigh the importance of certain results w.r.t. others? It didn’t seem like that based on the described results, but it would be helpful for the reader to clearly state the reason for reporting “study quality” scores. I guess that the authors just intended to provide some results on study quality distributions in this specific field?

Response: We did not include studies of weak quality in meta-analysis. We have added a sentence in paragraph 2 in the methods to clarify this (line 235-236 methods). However, we did report on these studies in the narrative synthesis. Reporting on study quality enabled us to provide an overall assessment of study quality and to identify particular areas of weakness for future recommendations. From assessment of study quality we identified the lack of use of validated measures for measuring alcohol use and the use of non-probability sampling methods as frequent limitations which is reported on in our results and discussion (results lines 280-285, discussion lines 209-219).

(4) A brief overview of the Higgins statistic would be helpful for the reader. How should small and large values of l^2 be interpreted? I also recommend that the authors add the corresponding reference in their manuscript (<https://www.bmj.com/content/327/7414/557>).

Response: We thank the reviewer for this feedback. We have added in a sentence defining the Higgins statistic and referencing this in the manuscript (line 252-255, methods).

Minor issues:

“weak quality .” → “weak quality.”

Response: Thank you this has been amended.

“Figure 1.” → “Figure 1:” (inconsistent use of “:” and “.” in figure legends)

Response: Thank you, we have amended this.

## Reviewer #3:

Overall, this study summarized the results of 22 years of research well and drew good meta-analysis results.
Only minor details need to be corrected.

1. There is no need to indicate statistically non-significant results in the abstract
"Harmful alcohol use was significantly associated with inconsistent condom use (pooled unadjusted OR:1.31; 95%CI: 0.96-1.80)"
It is better to use RR 1.65 (1.01-2.67) among the contents of Table 4b for this content.

Response: We thank the reviewer for pointing this out. We have amended this in the abstract to report the RR for inconsistent condom use instead, which is significant (line 67, abstract).

2. All figure numberings from "Harmful alcohol use" on page 12 are incorrect. should be corrected
Figure 1 -> Figure 2
Figure 2 -> Figure 3
Figure 3 -> Figure 4
Figure 4 -> Figure 5
Figure 5 -> Figure 6
Figure 6 -> Figure 7
Figure 7 -> Figure 8
Figure 8 -> Figure 9
Figure 9 -> Figure 10

Response: This has been corrected.

Reviewer #4:

 This study is good and brings up important aspects on this key population. However, in the abstract, the authors mentioned that there is 'no quantitative synthesis', I would suggest they change that statement, to say 'to our knowledge' as the quantitative synthesis could exist elsewhere (grey literature etc.) but they are not aware of it.
Secondly, they mentioned that there was an association between alcohol use and inconsistent condom use (OR 1.31 (0.96 - 1.80). The CI passes through 1 and therefore the association is not significant.
There is a typing error where they said Tabe 1 rather than Table 1 under sample techniques.

Response: We thank the reviewer for their feedback. We have amended the abstract to state ‘to our knowledge’ (line 33, abstract). We have also amended the abstract to report the pooled RR (RR 1.65; 95%CI: 1.01-2.67) of cohort studies for inconsistent condom use (line 67, abstract) which was significant, rather than the pooled OR.

Reviewer #5:

Comments for authors
This study was very well researched and written and clearly outlines the methodology for inclusion, quality of studies included, as well as the major findings. The study is clearly an important one. A major conclusion, about the fact that interventions are usually targeted at the individual level, rather than the environmental level, was noted in the Conclusion. Perhaps just a few more lines could be added about this very important finding, with perhaps a few lines added in the Introduction to discuss the syndemic nature of this phenomenon.

Response: We thank the reviewer for this feedback. We have amended the introduction discussion and conclusion to include syndemic theory so that this topic is covered throughout (line 148-153, introduction and lines 164-183, discussion).

General comments
Overall well-written, though several times authors used “which” instead of “that” – a quick read to correct would be useful.

Response: We have reviewed the manuscript and hopefully amended this grammatical point.

Specific comments
Abstract
In background, would have been helpful for authors to note why this topic is important. Overall, clearly written and informative.

Response: We have briefly included ‘which can lead to adverse health outcomes’ (line 30, abstract) in the abstract background to indicate the importance of this topic. Further detail on the adverse health outcomes of alcohol use is detailed in the introduction. Due to limited word count we have not elaborated on this further in the abstract.

Introduction
An important point made in the introduction – that since high income countries are implementing a range of rules/restrictions on alcohol, hence the need for alcohol companies to find replacement users – would have been impactful if noted in the Abstract.

Response: We thank the reviewer for this feedback but in order to keep the abstract succinct and within word count and to give space to focus on the results, we have not been able to add this into the abstract.

In the first paragraph, authors note the use pattern of alcohol use in sub-Saharan Africa. Would have been important to also include a comparison of what the use pattern is in high income countries.

Response: We have included a sentence on patterns of alcohol use in high income countries in the introduction (line 93-97, introduction).

Methods
Inclusion criteria – authors note they included studies that included FSW as participants, even if FSW were not the main focus of the study. How did they parse out the findings specifically for the FSW? Or did authors only include studies where the original authors already separated out findings for FSW?

Response: We included studies only where findings were separated out for FSWs. However, FSWs did not need to be the main focus of the study. For example, Ochonye (2019) study examining sexual risk behaviours in key populations included Female Sex Workers, Male Sex Workers and People With Injecting Drug Use but separated out results for each group (11).

- Data extraction
Line 6 - Meta-analysis should be changed to meta-analyses

Response: This has been amended.

Results
- Harmful alcohol use and STIs
Third paragraph – authors note that several studies reported on associations between alcohol use and ART adherence and viral suppression, however it is not clear what the direction of association was, i.e., positive or negative association. The associations were more clearly explained with PrEP use.

Response: We have included details on the direction of association in the results (lines 42-45, results).

Discussion
In the Results, authors noted that there was very high use of alcohol during first sex work. Would have been interesting, though not necessary, to add a bit of detail about this in the Discussion.

Response: We have added two sentences to the discussion addressing the implications of this (lines 124-127, discussion).

Reviewer #6:

This paper discusses the prevalence of harmful alcohol use among FSWs in LMICs and its associations with health and social concerns like violence/police arrest, condom use, HIV/STIs, drug use and mental health problems.
Minor comments:
1. The title of the paper does not accurately reflect the topic covered.
2. Have you thought about including grey literature?

Response: We thank the reviewer for their feedback.

1. We have amended the title to include ‘associated risk factors’.

2. We included studies from ‘citation list searching’. Although we did consider including grey literature we decided it was important for included studies to be peer-reviewed and so restricted our inclusion criteria to studies that were found in peer-reviewed journals.

Reviewer #7:

 Research Topic : Please remove the hyphen between "low" and "and"

Response: Previously published studies have used a hyphen in the term “low- and middle-income countries”(1) so we have kept to this standard.

Abstract:
Background. How should the first statement be written? I guess it should be "low and middle income countries" Please double check and kindly revise.

Response: Please see above, we have followed previously used grammar standards from published work.

Methods: case series analysis and experimental studies were captured only in the abstract and never mentioned again. Kindly check and make the necessary changes.

Response: Case series and experimental studies are mentioned in the abstract and the methods (lines 201-203, methods) of the manuscript. These are study designs that were part of our inclusion criteria, making them necessary to report on in the abstract and methods. In addition we report on baseline findings from experimental studies in our results.

Conclusion: your inclusion criteria means you did not consider longitudinal studies. You can therefore not make a conclusion that 'there is limited longitudinal studies based on your study.

Response: Our inclusion criteria state “The following study designs were included: cross-sectional survey, case–control study, cohort study, case series analysis, or experimental study with baseline measures for alcohol use.” Cohort studies are a type of longitudinal study which are part of our inclusion criteria. We report on findings from cohort studies in our results (lines 82-53 and table 3 and 5, results). Therefore, we can make conclusions on the availability of longitudinal studies in our conclusions.

Introduction: Do not use this "()" to enclose the intext reference. Please use this instead "[]". it makes recognition of intext reference difficult.

Response: This has been amended.

Paragraph 2: Please cite a reference for the sentence beginning with 'Sociocultural and economic factors...........

Response: This has been referenced.

Paragraph 4 Line 1 Please reconsider reframing the sentence ...... but there is generally............

Response: We have re-worded this sentence.

Methods:
Inclusion criteria: Was the year of publication of the selected papers included. please indicate it under this section

Response: We searched databases from first record until 24.02.2021. Year of publication is included in table 1.

Quality assessment: Elaborate on how the scoring was done. How did you score "yes", "No" and "cannot tell" and how did you arrive at the overall score for each article.

Response: Please see appendix 2 for CEBMa quality appraisal tool questions. We have detailed in our methods how we conducted the scoring (lines 223-236, methods) and have included additional details.

NB: None of the appendices is labelled A. I guess you mean appendix 1 but appendix 1 does not contain the information you are referring readers to. its rather the 3rd appendix. Kindly check and correct.

Response: Appendices have been reviewed and correctly labelled.

Results: How many of the studies were quantitative and how may employed mixed methods.
You repeatedly stated 'we included 92 cross sectional studies and 3 cohort studies but these information (study designs) were not captured in any of the tables. Please include the study designs in Table 1.

Response: We thank the reviewer for this feedback and have included the study design column of table 1. We also apologise for an error in reported study designs. Eighty-eight studies used cross-sectional designs to report on alcohol use, while six used a cohort design. and four experimental studies reported an alcohol intervention We have amended this in the results (results, line 288-292).

How many studies satisfied the inclusion criteria and were included. It looks like table 4b is reporting on 116 studies instead of the 98 you previously mentioned in the abstract and the method section.

Response: We are unclear where the reviewer is finding 116 studies in table 4b. Table 1 reports on 98 papers.

What is the "all" in table 4a and 4b indicating? please provide an interpretation in the results section.

Response: This is all studies reporting on a particular associated risk factor (e.g. violence) and was included to differentiate from the row below which excludes studies which selected FSWs based on HIV status. To avoid confusion we have removed ‘all’ from the table. We decided to report results for all eligible studies and excluding those which selected FSWs based on HIV status to address the risk of bias (lines 287-290, results and lines 197-198, discussion).

Limitation: Your search for articles did not prioritize or consider longitudinal studies. You can therefore not make a categorical statement that "there was lack of longitudinal studies". You should rather state the reality which is your study did not consider longitudinal studies.

Response: Our inclusion criteria Included cohort studies which are a type of longitudinal study. We report on findings from cohort studies in our results and therefore can make conclusions on the availability of longitudinal studies in our conclusions.

General comments: Some sentences were too long. it makes reading not interesting, causes reader fatigue and tend to loose its meaning at the tail end. otherwise it is a good publishable study.

Response: Thank for this feedback. Many of the sentences have been edited based on reviewer feedback. We hope this has improved the readability of the manuscript.

1.	Beattie TS, Smilenova B, Krishnaratne S, Mazzuca A. Mental health problems among female sex workers in low- and middle-income countries: A systematic review and meta-analysis. PLOS MEDICINE. 2020;17(9).

2.	Mueller M, D’Addario M, Egger M, Cevallos M, Dekkers O, Mugglin C, et al. Methods to systematically review and meta-analyse observational studies: a systematic scoping review of recommendations. BMC Medical Research Methodology. 2018;18(1):44.

3.	Baral S, Beyrer C, Muessig K, Poteat T, Wirtz AL, Decker MR, et al. Burden of HIV among female sex workers in low-income and middle-income countries: a systematic review and meta-analysis. LANCET INFECTIOUS DISEASES. 2012;12(7):538-49.

4.	Beattie TS, Krishnaratne S, Smilenova B, Mazzuca A. Mental health problems among female sex workers in low- And middle-income countries: A systematic review and meta-analysis. PLoS Medicine. 2020;17(9):1003297.

5.	Johnston LG, Sabin K. Sampling Hard-to-Reach Populations with Respondent Driven Sampling. Methodological Innovations Online. 2010;5(2):38-48.

6.	Heckathorn DD. Respondent-Driven Sampling: A New Approach to the Study of Hidden Populations*. Social Problems. 1997;44(2):174-99.

7.	Johnston LG, Chen YH, Silva-Santisteban A, Raymond HF. An Empirical Examination of Respondent Driven Sampling Design Effects Among HIV Risk Groups from Studies Conducted Around the World. AIDS AND BEHAVIOR. 2013;17(6):2202-10.

8.	Johnston LG, McLaughlin KR, El Rhilani H, Latifi A, Toufik A, Bennani A, et al. Estimating the Size of Hidden Populations Using Respondent-driven Sampling Data Case Examples from Morocco. EPIDEMIOLOGY. 2015;26(6):846-52.

9.	Sabin K, Hien MT, Huong PT, Johnston LG. Assessment of respondent driven sampling for recruiting female sex workers in two Vietnamese cities: Reaching the unseen sex worker. Journal of Urban Health. 2006;83(7 SUPPL.).

10.	Page MJ, McKenzie JE, Bossuyt PM, Boutron I, Hoffmann TC, Mulrow CD, et al. The PRISMA 2020 statement: an updated guideline for reporting systematic reviews. BMJ. 2021;372:n71.

11.	Ochonye B, Folayan MO, Fatusi AO, Bello BM, Ajidagba B, Emmanuel G, et al. Sexual practices, sexual behavior and HIV risk profile of key populations in Nigeria. BMC PUBLIC HEALTH. 2019;19(1).
